# Parenteral Nutrition as a Source of Xenobiotic Exposure in Preterm Infants in a Single-Center Case Study

**DOI:** 10.1021/acs.est.6c12407

**Published:** 2026-10-19

**Authors:** Michael Eigenschink, Nika Wattaul, Sabrina Berger, Jesse Peach, Ernst W. Müllner, Angelika Berger, Benedikt Warth, Harald H. Sitte, Lukas Wisgrill

**Affiliations:** † Department of Pediatrics, Division of Neonatology, Pediatric Intensive Care and Neuropediatrics, 27271 Medical University of Vienna, Waehringer Guertel 18-20, Vienna 1090, Austria; ‡ Comprehensive Center for Pediatrics, Medical University of Vienna, Waehringer Guertel 18-20, Vienna 1090, Austria; § Department of Food Chemistry and Toxicology, 27258 University of Vienna, Waehringer Strasse 38, Vienna 1090, Austria; ∥ Center for Medical Biochemistry, Max Perutz Laboratories, Medical University of Vienna, Dr.-Bohr-Gasse 9, Vienna 1030, Austria; ⊥ Exposome Austria, National Research Infrastructure and EIRENE-AT Node, Vienna 1090, Austria; # Center for Physiology and Pharmacology, Institute of Pharmacology, 27271 Medical University of Vienna, Waehringer Strasse 13a, Vienna 1090, Austria; ∇ Hourani Center for Applied Scientific Research, Al-Ahliyya Amman University, Amman 19328, Jordan; ○ AddRess Center for Addiction Research and Science, Medical University of Vienna, Waehringer Strasse 13a, Vienna 1090, Austria

**Keywords:** neonatal intensive care unit, prematurity, parenteral nutrition, exposomics, plasticizer, endocrine-disrupting chemicals, early life exposure

## Abstract

In premature infants, successful ex utero maturation depends on endogenous and exogenous stimuli. Chemical insults during this period may impact long-term development. Given immature hepatic biotransformation, parenteral xenobiotic exposure may be of particular toxicological relevance. We conducted a single-center case study to characterize the xenobiotic burden of neonatal parenteral nutrition (PN). A standardized PN regimen for a 28-week, 1000 g reference infant was passed through infusion lines and the flow-through analyzed via a multianalyte exposome LC-MS/MS assay. Eighteen of 221 targeted xenobiotics were detected. Diisononyl phthalate (DINP, 780 ng/mL), bisphenol A (BPA, 250 ng/mL), methylparaben (MP, 74 ng/mL), and 5-hydroxymethyl-2-furanoic acid (HMFA, 43 ng/mL) showed the highest mean concentrations. The estimated daily intake of BPA (9.5 μg/kg bw/d) exceeded calculated and official Health-Based Guidance Values. Consistent with immature detoxification, pharmacokinetic modeling suggested accumulation of DINP, MINP, and free BPA in premature infants. The higher predicted exposure of premature infants to free BPA warrants investigation into 4-methyl-2,4-bis­(4-hydroxyphenyl)­pent-1-ene (MBP) formation under potentially saturated Phase II metabolism. An exploratory analysis of plasma data from extremely premature infants indicated PN as a contributory rather than predominant source of intervention-related exposure. These findings represent a relevant clinical reference scenario rather than a population-wide exposure estimate.

## Graphical abstract

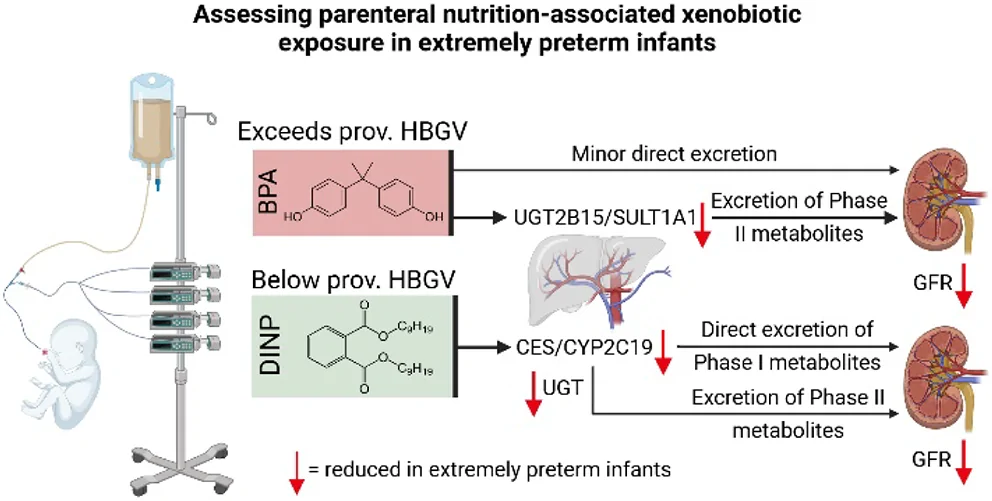

## Introduction

Extremely premature infants, especially those born with less than 1000 g body weight, represent a vulnerable patient population requiring a wide array of invasive treatments. Large observational cohort studies indicate that 84% of infants born between 22 and 28 weeks of gestation require invasive mechanical ventilation, 80% receive at least one red blood cell transfusion, and nearly all are treated with parenteral nutrition (PN, median duration = 17 days).^1^ Since key developmental processes in preterm infants occur ex utero, nonphysiological chemical exposures from medical devices coincide with a critical window of maturation.^2^ These early life exposures have the potential to impact development and may contribute to adverse long-term health outcomes.

Biomonitoring studies suggest considerable exposure of premature infants to plasticizers, most commonly phthalates.^3–5^ Plasticizers are chemical additives that are noncovalently embedded within the polymer matrix of plastics to impart favorable physicochemical properties. Polypropylene, polyethylene, polystyrene, and plasticized polyvinyl chloride are widely used in the manufacture of personal protective equipment, as well as patient contact devices such as infusion tubing, respiratory devices, blood bags, and liquid storage systems for medications.^6^ A recent study conducted in a surgical unit in New York demonstrated that 85% of tested medical devices (*n* = 46) leached at least six phthalates, and 98% of devices leached di­(2-ethylhexyl) phthalate (DEHP).^7^ Due to their endocrine-disrupting and potentially reprotoxic effects, several phthalates, including DEHP, were reclassified as “substances of very high concern” by the European Chemicals Agency (ECHA) and included in Annex XIV (REACH, 1907/2006).^8^ This subjects their use within the European Union (EU) to authorization requirements. Additionally, the EU Medical Device Regulation (2017/745)^9^ requires a justification/benefit-risk assessment (BRA) for recertification of (legacy) medical devices containing endocrine-disrupting substances or substances classified as CMR (carcinogenic, mutagenic, or toxic for reproduction) 1A/1B above 0.1% w/w. While desirable, this change may have encouraged heterogeneous substitution of substances used in medical devices, increasing the need for timely, clinically guided investigations of exposures.

Although studies have identified respiratory support and blood product administration as primary routes of exposure to plasticizers in premature infants,^3^ recent reports also highlighted that formulations for parenteral^10^ and enteral^11^ nutrition of infants contain xenobiotics. Common high-molecular-weight plasticizers preferentially partition into lipid emulsions,^12–14^ whereas low-molecular-weight, more polar plasticizers distribute into aqueous solutions.^14^ Thus, PN, comprising both lipid and aqueous fractions, may sustain a continuous influx of hydrophilic and lipophilic xenobiotics that are not subjected to hepatic first-pass metabolism. Our investigation of the plasma exposome of extremely premature infants with a gestational age (GA) of <28 weeks revealed the presence of several phthalates, parabens, and bisphenols, as well as significant amounts of 5-hydroxymethyl-2-furanoic acid (HMFA; ∼500–10,000 nmol/L).^5^ Since HMFA is predominantly formed via Maillard reactions from glucose during heat sterilization,^15^ exposure is likely to originate from parenteral nutrition. Consequently, its presence in plasma samples of premature infants suggests that continuous PN administration may contribute to overall xenobiotic exposure.

To clarify the contribution of PN to xenobiotic exposure in extremely premature infants, we simulated the parenteral regimen administered at our neonatology center. Specifically, we aimed to characterize the contaminant profile of neonatal PN, to determine whether estimated daily intakes of PN-associated xenobiotics exceed Health-Based Guidance Values (HBGVs), and to model whether the contaminants or their metabolites may accumulate under conditions of immature hepatic biotransformation and renal elimination. Our analyses identified DINP and BPA as the most abundant contaminants, with BPA intake exceeding established official as well as prematurity-adjusted provisional HBGVs. Pharmacokinetic modeling indicated that DINP, its metabolite MINP, as well as free BPA accumulate in premature infants relative to adults. Finally, an exploratory reanalysis of plasma data from premature infants supported the clinical detectability of PN-associated exposure through circulating HMFA, while indicating that PN contributes to, rather than dominates, the broader intervention-related xenobiotic burden.

## Materials and Methods

### Chemicals, Solvents, and Reagents

Chemicals, solvents, and reagents were of LC-MS grade unless stated otherwise. Detailed information on the exogenous compounds used in the LC-MS analysis is provided in the Supplementary Table S1. Analytical standards for calibration and method performance evaluation were obtained as a comprehensive multistandard solution comprising diverse chemical classes, including plasticizers, bisphenols, parabens, per- and polyfluoroalkyl substances (PFAS), and other environmental contaminants.

### Experimental Setup to Simulate Parenteral Nutrition

PN was simulated using routinely employed plastic medical devices and intravenous fluids required to provide full PN to extremely premature infants in our neonatal ward at the Medical University of Vienna. A full list of the devices and pharmaceutical products used to prepare the respective PN mixtures is provided in Table 1. The pharmaceuticals, solutions, infusion bags, administration sets, syringes, and needles/cannulas were provided by the clinical team for each experimental run and corresponded to those used in routine clinical practice on the respective day. A detailed list of the batch/lot numbers used in each independent experiment is provided in the Supplementary Table S2. Dosing and total PN volumes were calculated using CatoPAN (BD Biosciences) to generate a standardized infusion for a hypothetical 1000 g extremely premature infant. The scenario was selected instead of a more extreme edge-of-viability model because PN management in such cases is often highly individualized and influenced by clinical factors, including fluid requirements, hemodynamic instability, and center-specific treatment strategies. The weight of 1000 g was used to normalize dosing and exposure estimates to per kilogram body weight, with a total calculated infusion volume of 160 mL/kg bw/d. Routine total PN consists of three components: (1) a water-based glucose solution at a clinically guided concentration, (2) an amino acid formulation with electrolytes and trace elements, and (3) a lipid solution with vitamins. The flow rates for the individual solutions were defined as follows: glucose 3.38 mL/h, amino acids 2.11 mL/h, and lipids 0.87 mL/h. Since the glucose component is routinely diluted with sterile water, its final concentration varies based on the clinical target for fluid intake. Given this nonstandardizable practice, analyses focused on the more stable components: the amino acid–based admixture (hereafter “aqueous mixture”) and the lipid emulsion (hereafter “lipid mixture”). Continuous infusion was simulated using a syringe pump system with respective infusion lines. Samples were collected directly from the lines after 0, 4, 12, and 24 h of administration, as illustrated in Figure 1A. A total of four independent experiments were performed on three different experimental days across a time span of 4 weeks. For each independent experiment, one aqueous mixture and one lipid mixture were prepared anew, passed through the respective infusion lines, and sampled at 0, 4, 12, and 24 h. Aqueous and lipid samples from the same experiment are therefore paired. The setup yielded a total of 32 samples (four independent experiments for two mixture types sampled across four time points), consisting of 16 aqueous and 16 lipid samples. The aqueous and lipid mixtures were stored at −80 °C until further analysis.

**Table 1:** Plastic Medical Ware and GMP-Grade Solutions Used for the Preparation of the Aqueous and Lipid PN-Mixture^a^ ^b^

Plastic ware				
Product	Company	Material	Pack	Label
Braun Injekt Luer Solo syringe	B. Braun	PrP/PE	5 mL	BPA-/PVC-/DEHP-free
Braun Injekt Luer Solo syringe	B. Braun	PrP/PE	20 mL	BPA-/PVC-/DEHP-free
BD Blunt Fill Needle 18G × 1 1/2″	BD	metal	n.a.	n.a.
Braun Original Perfusor Syringe	B. Braun	PrP/PE	50 mL	BPA-/PVC-/DEHP-free
Infusion line with 0.2 μm filter	Impromediform	PU	n.a.	DEHP-free
Infusion line with 1.2 μm filter light-protected	Impromediform	PU	n.a.	DEHP-free
Infusion bag EVA	Impromediform	EVA	150 mL	DEHP-free
Transfer set with automatic valve, light-protected	Impromediform	PU	n.a.	PVC-/DEHP-free

Solutions				
**Product**	**Company**	**Material**	**Pack**	**Label**
Primene 10% pure	Baxter Healthcare	glass	100 mL	n.a.
Potassium-l-malate 1 mol/L	Internal pharmacy	PrP	50 mL	n.a.
Calcium gluconate 10%	B. Braun	PE	10 mL	PVC-/DEHP-free
Calcium Fresenius	Fresenius Kabi	glass	10 mL	n.a.
Sodium glycerophosphate 306.1 mg/mL	Fresenius Kabi	PE	10 mL	n.a.
Glucose-1-phosphate 1 mol/L	Fresenius Kabi	glass	10 mL	n.a.
Sodium chloride 0.9%	B. Braun	PE	10 mL	PVC-/DEHP-free
Sodium chloride 1 mol/L	Fresenius Kabi	PrP	20 mL	n.a.
Magnesiocard i.v.	Verla-Pharm	glass	10 mL	n.a.
Peditrace	Fresenius Kabi	PrP	10 mL	n.a.
SMOFlipid 200 mg/mL emulsion	Fresenius Kabi	glass	100 mL	n.a.
Soluvit	Fresenius Kabi	glass	10 mL	n.a.
Vitalipid	Fresenius Kabi	glass	10 mL	n.a.

a PrP = polypropylene, PE = polyethylene, PU = polyurethane, EVA = ethylene vinyl acetate.

b n.a. = not available/applicable.

**Figure 1:**
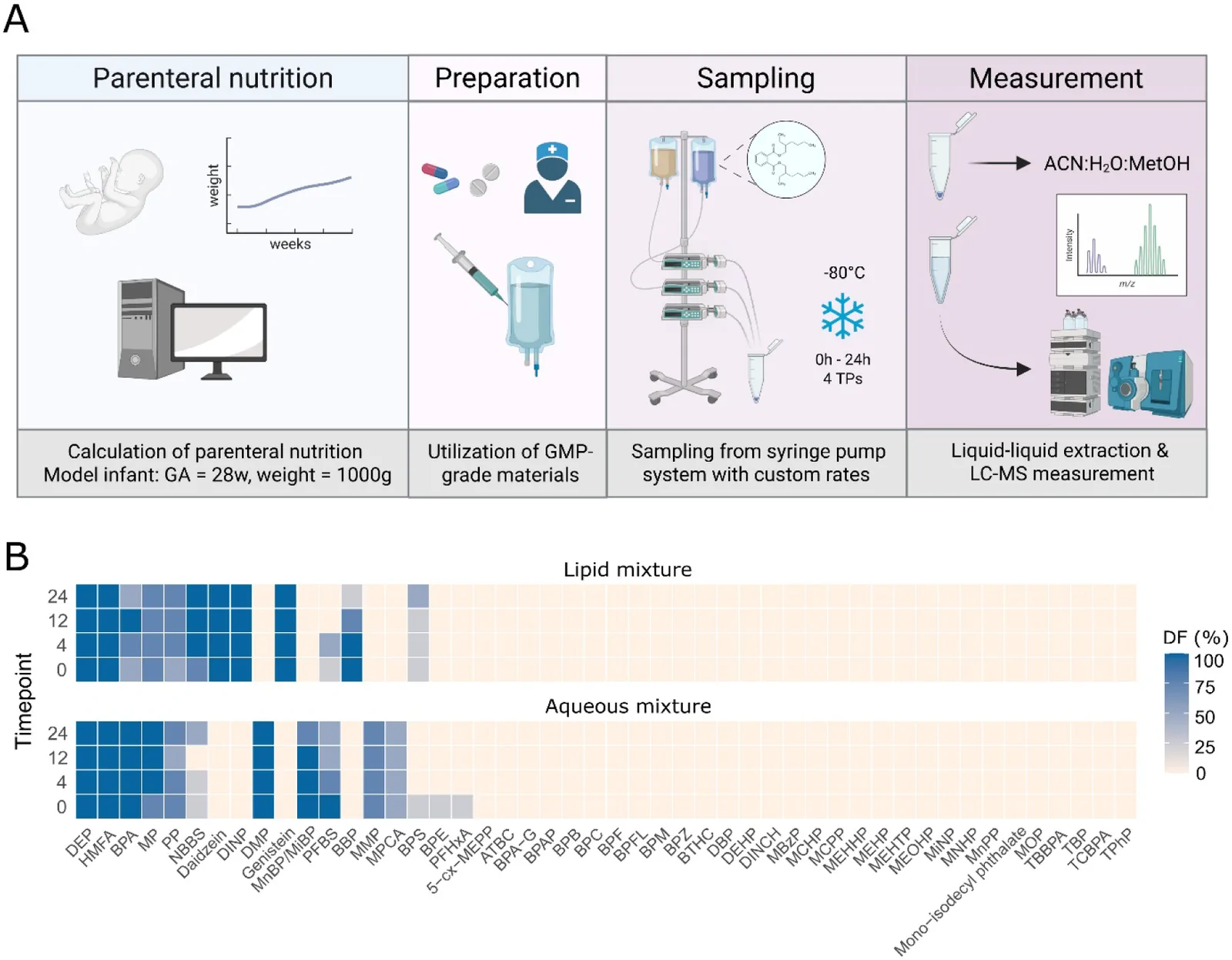
Experimental workflow and detection frequency of analytes in simulated parenteral nutrition. (A) Schematic overview of the study design. A standardized parenteral nutrition dosing regimen was calculated for an extremely preterm infant (GA = 28 weeks, birthweight = 1000 g). Parenteral nutrition was prepared using GMP-grade solutions and routine medical devices prevalent at a NICU located at the Medical University of Vienna. Four independent experiments were performed, each of which comprised an aqueous and lipid mixture. During each experiment, samples were taken from the distal end of the respective infusion line at 0, 4, 12, and 24 h, yielding a total of 32 samples (*n*_(aq, 0–24h)_ = 16, *n*_(lip, 0–24h)_ = 16). Samples were stored at −80 °C until LC-MS/MS analysis. (B) Heatmap depicting the per-time point detection frequency (DF, %) of the measured compounds for the lipid (top) and aqueous mixture (bottom). DF represents the percentage of positive detections for a compound across four independent experiments sampled at a given time point (0 h, 4 h, 12 h, 24 h) for a given solution (*n* = 4). Infographics were created with BioRender.com.

### Sample Preparation and Analysis

Aqueous (*n*_(0–24h)_ = 16) and lipid mixture (*n*_(0–24h)_ = 16) samples were thawed and vortexed. A dilute-and-shoot approach was applied for analysis. Aqueous aliquots (135 μL) were diluted with 15 μL acetonitrile (ACN), and lipid aliquots (75 μL) were diluted 1:1 (v/v) with ACN. Different dilutions were chosen to account for matrix effects and ensure robust, reproducible analysis. The diluted samples were vortexed, centrifuged at 18,000 × *g* for 5 min at 4 °C, and the resulting supernatants were transferred into LC-MS vials with glass inserts. Quantification was performed using a 10-point calibration curve with target analytes diluted in ACN:H_2_O (1:1, v/v). The final concentrations of each analyte at the individual calibration levels are provided in the Supplementary Table S3. Spiked matrix samples were prepared in triplicate for each matrix by pooling 0-h control samples and spiking them at a single concentration level. The final analyte concentrations in the spiked matrix samples are reported in the Supplementary Table S3. Laboratory process blanks were prepared in triplicate by replacing the matrix samples with LC-MS-grade water. Instrument performance was constantly monitored and spiked matrix samples were analyzed in triplicate per matrix to assess repeatability.

### LC-MS/MS Instrumentation and Parameters

Targeted analyses were performed on an LC-MS/MS system operated in scheduled multiple reaction monitoring mode with fast polarity switching. The system consisted of a 1290 Infinity II UHPLC (Agilent) coupled to a QTRAP 7500 (Sciex) mass spectrometer with an electrospray ionization (ESI) source. The analytical method was based on the LC-MS/MS assay developed by Preindl et al.^16^ and adapted from the extended and validated quantitative exposomics workflow by Gu et al.^17^ Chromatographic separation was achieved using a reversed-phase Acquity UPLC HSS T3 column (1.8 μm, 2.1 × 100 mm, Waters) equipped with a VanGuard precolumn (1.8 μm, Waters). Mobile phases consisted of water with 0.3 mM ammonium fluoride (eluent A) and acetonitrile (eluent B). The column oven and autosampler temperatures were set to 40 and 7 °C, respectively. The flow rate was 0.4 mL/min, and the injection volume was 5 μL. The gradient program (Table S4) started at 5% eluent B and was held for 1 min, followed by a linear increase to 18% B at 1.8 min and to 35% B at 4.2 min. Eluent B was then increased to 48% at 13.0 min and subsequently raised to 90% at 15.8 min. A high-organic wash was performed by increasing eluent B to 98% at 15.81 min and to 100% at 19.0 min, held until 22.5 min. The system was then returned to initial conditions (5% B) at 22.6 min, followed by a re-equilibration step until 25 min. Compound-specific multiple reaction monitoring transitions and retention times are provided in the Supplementary Table S5. The source temperature was 500 °C, the curtain gas 45 psi, and ion source gases 1 and 2 were 50 psi. The ion spray voltage was set to 2500 V in positive mode and −2500 V in negative ionization mode.

### Quality Control and Data Processing

Data were acquired in SciexOS (3.4) and processed in Skyline (v24.1.0.414). Peak integration was visually inspected and manually adjusted to ensure consistent peak boundaries for each peak. Linear calibration curves with 1/x weighting were fitted to the solvent-based calibration data. Analyte concentrations in samples were calculated from the calibration curves and corrected for dilution factors. Calibration curve acceptance required a coefficient of determination (*R* ^2^) ≥ 0.95. *R* ^2^ values and calibration ranges are provided in the Supplementary Table S6. For analytes detected in process blanks (Table S6), blank subtraction was performed by subtracting the maximum concentration observed in process blanks from all sample concentrations. Matrix effects were assessed by determining signal suppression or enhancement (SSE). SSE was calculated for each analyte as the ratio between the peak area measured in the spiked matrix sample and the peak area of the solvent-based calibration standard at the same concentration. Measured sample concentrations were corrected for matrix effects using the corresponding SSE values. Limits of detection (LOD) and limits of quantification (LOQ) were estimated based on signal-to-noise (S/N) ratios determined in spiked matrix samples. The LOD was set as an S/N ratio >3, while the LOQ was defined as the lowest concentration yielding an S/N ratio >10. Analyte signals detected between LOD and LOQ were reported accordingly.

### Analysis of Exposure Data

All compounds detected above the LOD in at least one sample were retained for subsequent analyses. Detection frequency for a given compound was expressed as the ratio between samples in which the compound was detected above the LOD and the total number of samples. For numerical calculations, values below the LOD were imputed as LOD/2, whereas values below the LOQ but above the LOD were substituted with (LOQ+LOD)/2. Compounds detected in >30% of samples in at least one solution were selected for kinetic analyses. LOESS smoothing was used to approximate time-dependent changes in exposure. For analysis of cumulative xenobiotic exposure over 24 h, the area under the concentration–time curve (AUC_(0–24h)_) of relevant compounds was computed by the trapezoid method using the DescTools package.^18^ To account for time-dependent changes in xenobiotic exposure in our pharmacokinetic models, mean BPA_(aqueous)_ concentrations were approximated by nonlinear least-squares regression using a hyperbolic decay function *y*_(*t*)_ = *y*_0_ + (*A*/(*t*+*c*)). Mean DINP_(lipid)_ concentrations were modeled using ordinary least-squares regression with the quadratic polynomial *y*_(*t*)_ = β_0_ + β_1_*t + β_2_*t ^2^. Analyses were restricted to the measurement interval (0–24 h) to avoid extrapolation artifacts. For BPA_(lipid)_, the mean of concentrations across all time points was used due to flat kinetics (absence of time-dependency). No calculations were performed for DINP_(aqueous)_ as the compound was not detected in the aqueous solution.

### RNA-Seq Analysis of Phase I and II Metabolism

For the analysis of high-temporal-resolution hepatic gene expression in mice, FPKM values were retrieved from Gene Expression Omnibus (GEO, accession number *GSE58827)*.^19^ For the cross-species comparison of hepatic Phase I and Phase II enzyme expression over maturation, CPM values were accessed from ArrayExpress for *Homo sapiens* (*E-MTAB-6814*), *Macaca mulatta* (*E-MTAB-6813*), *Mus musculus* (*E-MTAB-6798*), and *Rattus norvegicus* (*E-MTAB-6811*).^20^ Entries were matched to gene symbols for the respective species using AnnotationDbi. The orthogene package^21^ was used to perform orthologous gene mapping via the babelgene database. For many-to-one relationships, the human gene equivalent was modeled as the mean of multiple entries. One-to-many relationships were not restricted, allowing gene expression to be assigned to multiple corresponding human orthologs. Only genes with nonzero values in more than 75% of samples were retained for analyses. For high-resolution mouse data, remaining zero entries were substituted with half of the minimum expression of the respective gene. Data sets were filtered for genes relevant to Phase I and Phase II biotransformation. The ComplexHeatmap package^22^ was used for hierarchical clustering of log2-transformed, z-scaled granular mouse data. Time-resolved relative expression profiles were computed for each gene family species combination by normalizing the mean expression of each gene at a given time point to its cumulative expression across all time points and then averaging the normalized values across genes belonging to the same gene family. For cross-species comparisons, developmental stage alignment was performed using the data provided by Cardoso-Moreira et al.^20^ Direct comparison between neonatal and adult hepatic tissue gene expression was performed using CPM values from selected maturation stages.

### Pharmacokinetic Modeling of Xenobiotic Exposure

Constant-rate infusion, one-compartment, first-order elimination pharmacokinetic models were created for DINP and BPA using mrgsolve (Version 1.6.1).^23^ An extensive literature search was performed to (i) identify metabolic pathways associated with compound elimination, (ii) derive the plasma half-life and volume of distribution for parent compounds and metabolites, and (iii) identify hepatic Phase I and Phase II enzymes responsible for compound oxidation and conjugation. Whenever possible, data from intravenous application of substances into humans or primates were used to inform modeling decisions. Compound clearance was scaled for preterm infants based on published data on differences in hepatic volume,^24,25^ hepatic enzyme expression,^26–29^ and renal filtration^30,31^ between adults and infants. As the summary of evidence underlying modeling decisions and equations exceeds the scope of this section, comprehensive descriptions for each model have been added to the Supplementary Document (Chapters S1 and S2). Following mathematical compilation, real-world data on infusion rates of aqueous- and lipid PN, as well as daily body weight, were extracted from the clinical data management system for a premature infant (GA = 28 weeks, birthweight = 1000 g). These data were then used in the models to correctly account for changes in infusion rates and birthweight over time. Moreover, time dependence of substance release during PN was modeled by fitting continuous functions to mean concentrations measured at 0, 4, 12, and 24 h for aqueous and lipid-mixtures, respectively. The pharmacokinetic model was simulated at hourly resolution. Finally, to assess the effects of prematurity on pharmacokinetics, the full model (preterm, GA = 28 weeks, birthweight = 1000 g) was compared to a reduced model (full model without enzyme scaling) and an adult model (70 kg reference adult).

### Analysis of Parenteral Nutrition-Associated Exposures in the NICU-Setting

Absolute concentrations of selected xenobiotics in the plasma of premature infants quantified in our previous study by Jamnik et al.^5^ were reanalyzed after stratification by ongoing parenteral nutrition at the time of blood sampling. Collection of blood and clinical data was approved by the Ethics Committee of the Medical University of Vienna (No. 1348/2017). Consent was obtained from the parents or legal guardians of all included infants. Wilcoxon rank-sum tests were performed for the five analytes of interest (BBP, BPA, HMFA, MEHP, and NBBS), and *p*-values were adjusted for multiple testing using the Benjamini–Hochberg (BH) procedure (adj. *p*-value cutoff = 0.05). To assess potential confounding by red blood cell transfusion as a source of HMFA exposure, the analysis was repeated after excluding infants who had received a red blood cell transfusion within 3 days prior to blood sampling. For Pearson correlation analyses between HMFA and the other four xenobiotics, left-censored values were imputed as LOD/2 prior to log10 transformation, and *p*-values were adjusted using the BH procedure. Correlation analyses were initially restricted to infants receiving parenteral nutrition (PN) at the time of blood sampling and were subsequently repeated in the entire cohort as a sensitivity analysis.

### Calculation of Hazard Quotients

Toxicological points of departure (PoDs) were retrieved for each substance from documents published by recognized national and international regulatory authorities, including the European Medicines Agency (EMA), the European Food Safety Authority (EFSA), the ECHA, the Environmental Protection Agency (EPA), and the Australian Industrial Chemicals Introduction Scheme (AICIS). In accordance with ICH Q3C­(R9),^32^ provisional permitted daily exposures were derived by reviewing PoD-informing toxicological studies on a case-by-case basis and applying toxicological assessment factors (F1–F5) to the respective PoDs. Consistent with the 2025 draft ICH Q3E framework on extractables and leachables,^33^ an additional route-to-route extrapolation factor was applied, where required, to account for parenteral exposure. The resulting values were subsequently adjusted using a preterm-specific assessment factor to calculate provisional parenteral HBGVs for premature infants. For DINP and BPA, this factor was derived using an EFSA-informed approach^34^ by correcting the default toxicokinetic uncertainty factor of 3.16 with estimates obtained from the pharmacokinetic model via multiplication. Because substance-specific pharmacokinetic modeling for the remaining compounds was beyond the scope of this study, an additional 10-fold FQPA-based sensitivity factor was applied as a conservative default. A detailed description of the search strategy and extensive information on respective calculations on a per-substance basis is provided in Supplementary Chapter S3. Importantly, calculated values do not reflect official regulatory body decisions and should be regarded as provisional estimates rather than established regulatory HBGVs. Estimated daily intake (EDI) of xenobiotics via parenteral nutrition was calculated by multiplying AUC_(0–24h)_ values, derived from kinetic data using the trapezoidal method, with the respective syringe-pump flow rates (Rin_(lipid)_ = 0.87 mL/h, Rin_(aqueous)_ = 2.11 mL/h). Hazard quotients (HQs) were then estimated as the ratio of the summed EDI (lipid + aqueous) to the corresponding HBGV. HQ values >1 indicate exceedance of the respective HBGV.

## Results

### Exogenous Xenobiotic Contaminants in the Parenteral Nutrition of Premature Infants

Eighteen of 221 screened xenobiotics were detected (Figure 1B) using a real-world model of lipid- and water-based PN (*n*_(aq/lip, 0–24h)_ = 16; four independent experiments across four time points) for premature infants and employing GMP-grade products from our neonatal intensive care unit. Of these, only HMFA (median_(aq)_ = 42 ng/mL, median_(lip)_ = 3.6 ng/mL), a byproduct of heat sterilization, and the low-molecular-weight compound diethyl phthalate (DEP; median_(aq)_ = 0.64 ng/mL, median_(lip)_ = 0.83 ng/mL) were detectable across all time points in both solutions (*n*_(total)_ = 32). Other xenobiotics preferentially leached into either the lipid-rich (*n*_(0–24h)_ = 16) or aqueous solution (*n*_(0–24h)_ = 16), depending on side chain length and overall polarity. For example, DINP and dimethyl phthalate (DMP), two compounds with long and short acyl-chains, respectively, exhibited selective partitioning into the lipid emulsion (DINP, DF_(lipid, 0–24h)_ = 100%, median_(lip, 0–24h)_ = 660 ng/mL) and the aqueous solution (DMP, DF_(aq, 0–24h)_ = 100%, median_(aq, 0–24h)_ = 0.96 ng/mL). Monomethyl phthalate (MMP), the hydrolysis product of DMP, was present at higher concentration within the aqueous samples (DF_(aq, 0–24h)_ = 75%, median_(aq, 0–24h)_ = 4.0 ng/mL) than its parent compound. Butyl benzyl phthalate (BBP) and the chromatographically inseparable metabolites mono-*n*-butyl phthalate/monoisobutyl phthalate (MnBP/MiBP) also selectively contaminated lipid (DF_(BBP, 0–24h)_ = 75%, DF_(MnBP/MiBP, 0–24h)_ = 0%) or aqueous samples (DF_(BBP, 0–24h)_ = 0%, DF_(MnBP/MiBP, 0–24h)_ = 94%). Regarding bisphenols, BPA was present in all aqueous samples (DF_(aq, 0–24h)_ = 100%, median_(aq, 0–24h)_ = 8.2 ng/mL), albeit at lower concentrations compared to the lipid mixture (DF_(lip, 0–24h)_ = 69%, median_(lip, 0–24h)_ = 170 ng/mL). Bisphenol S (BPS) was detected in lipid mixtures (DF_(lip, 0–24h)_ = 31%) but was only minimally present in aqueous solutions (DF_(aq, 0–24h)_ = 6.3%). Bisphenol E (BPE) was found in one aqueous sample. The parabens methylparaben (MP) and propylparaben (PP) were detected in both mixtures, with higher concentrations in the aqueous compared to lipid PN (MP, median_(aq, 0–24h)_ = 60 ng/mL vs median_(lip, 0–24h)_ = < LOQ; PP, median_(aq, 0–24h)_ = 1.3 ng/mL vs median_(lip, 0–24h)_ = < LOQ). Perfluorobutanesulfonic acid (PFBS) was detected frequently in the aqueous mixture (DF_(aq, 0–24h)_ = 69%) and only occasionally in the lipid emulsion (DF_(lip, 0–24h)_ = 19%). Perfluorohexanoic acid (PFHxA) was detected in only one aqueous sample. The two isoflavones, genistein and daidzein, were selectively enriched in the lipid mixture (genistein, DF_(lip, 0–24h)_ = 100%, median_(lip, 0–24h)_ = 0.031 ng/mL vs daidzein, DF_(lip, 0–24h)_ = 100%, median_(lip, 0–24h)_ = 0.054 ng/mL), likely reflecting the soy basis of the emulsion. All data are summarized in Table 2.

**Table 2:** Xenobiotics Detected in Aqueous and Lipid PN Mixtures (*n* = 4 Independent Experiments) across All Time Points (*n*_(0–24h)_ = 16)^a^

		Aqueous mixture (*n*_(0–24h)_ = 16)						Lipid mixture (*n*_(0–24h)_ = 16)					
Substance category	Compound	LOD (ng/mL)	LOQ (ng/mL)	DF (%)	Mean (ng/mL)	Median (ng/mL)	Range (ng/mL)	LOD (ng/mL)	LOQ (ng/mL)	DF (%)	Mean (ng/mL)	Median (ng/mL)	Range (ng/mL)
Plasticizer	BBP	0.0096	0.032	0.0	<LOD	<LOD	<LOD	0.0058	0.019	75	0.18	0.15	<LOD – 0.75
	DEP	0.046	0.15	100	0.64	0.64	0.34 – 0.88	0.15	0.51	100	1.1	0.83	<LOQ – 2.8
	DINP	53	180	0.0	n.d.	n.d.	n.d.	69	230	100	780	660	<LOQ – 1300
	DMP	0.0077	0.026	100	1.1	0.96	0.42 – 2.0	0.068	0.23	0.0	n.d.	n.d.	n.d.
	MMP	0.78	2.6	75	3.8	4.0	n.d. – 10	2.9	9.6	0.0	n.d.	n.d.	n.d.
	MnBP/MiBP	0.079	0.26	94	0.34	0.39	<LOD – 0.60	0.076	0.25	0.0	<LOD	<LOD	<LOD
	NBBS	0.13	0.45	25	<LOD	<LOD	<LOD – <LOQ	0.27	0.91	94	1.1	<LOQ	<LOD – 4.2
Plastic additives	BPA	1.3 × 10^–4^	4.4 × 10^–4^	100	250	8.2	0.043 – 2400	0.0041	0.014	69	250	170	n.d. – 630
	BPE	0.029	0.098	6.3	<LOD	n.d.	n.d. – <LOQ	0.046	0.15	0.0	n.d.	n.d.	n.d.
	BPS	8.1 × 10^–4^	0.0027	6.3	0.0066	<LOD	<LOD – 0.099	0.0022	0.0072	31	0.12	<LOD	<LOD – 1.8
Personal care products	MP	3.1 × 10^–4^	0.0010	94	74	60	<LOD – 170	0.052	0.17	75	<LOQ	<LOQ	<LOD – 0.2
	PP	3.2 × 10^–4^	0.0011	69	10	1.3	n.d. – 76	0.0055	0.018	69	<LOQ	<LOQ	n.d. – 0.069
PFAS	PFBS	0.0011	0.0035	69	0.014	<LOQ	<LOD – 0.17	0.0027	0.0091	19	0.30	<LOD	<LOD – 4.1
	PFHxA	0.041	0.14	6.3	<LOD	n.d.	n.d. – <LOQ	0.093	0.31	0.0	n.d.	n.d.	n.d.
Maillard reaction	HMFA	0.76	2.5	100	43	42	37 – 51	0.68	2.3	100	3.3	3.6	<LOQ – 4.8
Isoflavones	Daidzein	0.0071	0.024	0.0	n.d.	n.d.	n.d.	0.0027	0.0089	100	0.055	0.054	0.038 – 0.076
	Genistein	0.0091	0.030	0.0	n.d.	n.d.	n.d.	0.0014	0.0048	100	0.032	0.031	0.019 – 0.048
Polyphenols	MPCA	0.0074	0.025	50	0.36	0.20	n.d. – 1.1	0.0057	0.019	0.0	n.d.	n.d.	n.d.

a Detection frequencies and summary statistics were calculated across all 16 samples per mixture irrespective of sampling time point (four independent experiments comprising an aqueous and lipid mixture, measured at four time points: 0, 4, 12, and 24 h). Thus, these pooled (overall) detection frequencies differ from the per-time point detection frequencies shown in Figure 1B. DF = Detection Frequency, LOD = limit of detection; LOQ = limit of quantification; n.d. = not determined. Values were rounded to two significant digits. Scientific notation was used for values <10^–3^ or >10^4^.

### Area-Under-The-Curve Analysis of Xenobiotic Exposure over 24 h

The area under the concentration–time curve (AUC_(0–24h)_) was calculated for each analyte and mixture to quantify the cumulative exposure over 24 h from continuous perfusion (Table 3). This metric estimates xenobiotic concentration over the 24 h sampling period and allows direct comparison between formulations despite differences in leaching kinetics (Figure 2A). The AUC_(0–24h)_ values (*n*_(AUC)_ = 4 per mixture type and compound) were calculated from concentration measurements obtained at 0, 4, 12, and 24 h for four independent experiments for each mixture type. Calculated AUC_(0–24h)_ values confirmed mixture-specific contamination across detected compound classes, with peak values for substances primarily found in one of the two solutions. In the lipid emulsion, DINP accounted for the highest xenobiotic exposure, followed by BPA and HMFA (Figure 2B). The aqueous solution contained significant amounts of BPA and HMFA, along with comparatively invariant levels of the hydrophilic phthalate DMP. The kinetics (Figure 2A) and data variance (Figure 2B) indicated that leaching of amphiphilic compounds, such as parabens, is associated with greater exposure variability. This might be explained by differences in the polymer-fluid partition coefficients across matrices. Six compounds detected in the parenteral infusion model were also identified in plasma samples from our previously published biomonitoring data set of 21 extremely preterm infants:^5^ BPA (DF_(plasma)_ = 100%), MP (DF_(plasma)_ = 21%), PP (DF_(plasma)_ = 100%), *n*-butylbenzenesulfonamide (NBBS; DF_(plasma)_ = 100%), BBP (DF_(plasma)_ = 100%), and HMFA (DF_(plasma)_ = 52%).

**Table 3:** Cumulative 24 h Exposure Estimates (AUC_(0–24h)_) in ng·h/mL for Detected Xenobiotics in Simulated Neonatal Parenteral Nutrition^a^

		Aqueous mixture (*n*_(AUC)_ = 4)		Lipid mixture (*n*_(AUC)_ = 4)	
Substance category	Compound	Mean AUC_(0–24h)_	Range AUC_(0–24h)_	Mean AUC_(0–24h)_	Range AUC_(0–24h)_
Plasticizer	BBP	0.12	0.12 – 0.12	4.1	3.3 – 5.7
	DEP	15	14 – 17	24	18 – 35
	DINP	0.0	0.0 – 0.0	2.2 × 10^4^	1.6 × 10^4^ – 2.5 × 10^4^
	DMP	26	11 – 35	0.0	0.0 – 0.0
	MMP	87	0 – 160	0.0	0.0 – 0.0
	MnBP/MiBP	8.1	3.3 – 11	0.91	0.91 – 0.91
	NBBS	2.7	1.6 – 3.4	27	13 – 60
Plastic additives	BPA	2200	430 – 5200	5600	3.8 – 1.3 × 10^4^
	BPS	0.059	0.0097 – 0.21	2.8	0.026 – 11
Personal care products	MP	1800	0.75 – 3600	2.3	0.62 – 3.2
	PP	110	0.037 – 220	0.42	0.050 – 1.1
PFAS	PFBS	0.32	0.020 – 1.1	6.5	0.032 – 25
Maillard reaction	HMFA	1000	930 – 1100	80	47 – 100
Isoflavones	Daidzein	0.0	0.0 – 0.0	1.3	0.99 – 1.6
	Genistein	0.0	0.0 – 0.0	0.76	0.52 – 1.1
Polyphenols	MPCA	8.9	0 – 18	0.0	0.0 – 0.0

a AUC_(0–24h)_ values (*n*_(AUC)_ = 4 per mixture type) were calculated from concentration measurements obtained at 0, 4, 12, and 24 h during four independent experiments comprising an aqueous and lipid mixture. AUC = area under the concentration–time curve, Range = min – max. Values were rounded to two significant digits. Scientific notation was used for values <10^–3^ or >10^4^.

**Figure 2:**
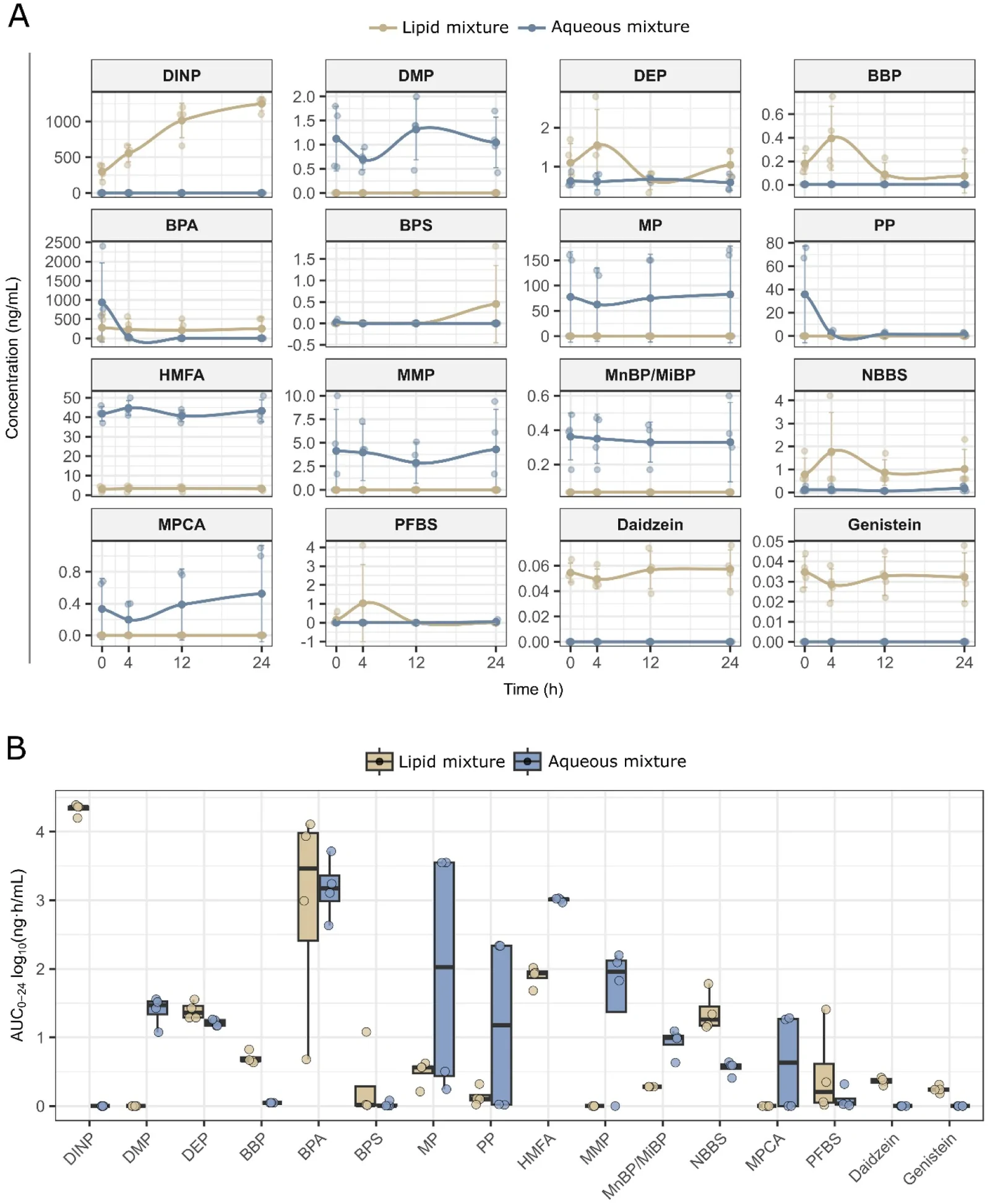
Xenobiotic release kinetics into parenteral nutrition and area under the concentration–time curve analysis (A) Concentration profiles of xenobiotics released into the lipid (beige) or aqueous (blue) fraction of neonatal parenteral nutrition measured at distinct time points (0 h, 4 h, 12 h, 24 h; *n* = 4, each). (B) Areas under the concentration–time curves calculated for each independent experiment (*n* = 4 per mixture) across all determined time points (0 h, 4 h, 12 h and 24 h) for both mixture types using the trapezoid method for numerical integration. Values are shown as boxplots for substances partitioning into the lipid (beige) and aqueous (blue) mixture.

### Hepatic Expression of Phase I and Phase II Enzymes across Ontogeny

The preceding analyses describe the cumulative xenobiotic exposure of infants receiving PN. The potential toxicity of xenobiotic compounds, especially when present in mixtures, however, is dependent on their biotransformation. Since intestinal metabolism is bypassed during PN, the detoxification of lipophilic compounds largely relies on hepatic Phase I and Phase II capacity. Due to the scarcity of developmental human data, high-temporal-resolution hepatic RNA-seq data from mice^19^ were reanalyzed and expression trajectories of biotransformation pathways compared with less temporally resolved human, rat, and rhesus macaque data sets.^20^ Hierarchical clustering of ortholog-matched hepatic RNA-seq data from mice sampled at 12 distinct time points showed shifts in the expression of Phase I and Phase II transcripts between gestational day 17.5 (GD 17.5) and day of life 60 (DoL-60, Figure 3A). These shifts involved several genes relevant to the biotransformation of substances detected in PN solutions (Figure 3B). Analysis of expression trajectories across enzyme families demonstrated pronounced changes in the abundance of alcohol dehydrogenase (ADH) and cytochrome P450 (CYP) genes as well as glutathione-S-transferase (GST) and uridine diphosphate glucuronosyltransferase transcripts between neonatal (GD 17.5/DoL-0) and adult (DoL-45 to DoL-60) mice (Figure 3C). These temporal changes were reproducible across mouse data sets (Figure 3D), with similar patterns observed in rats. Importantly, pronounced postnatal adaptations in the expression of enzyme families relevant to xenobiotic Phase I (carboxylesterases = CES, CYP) and Phase II (UGT) biotransformation of substances measured in the infusion solutions were also observed in humans (Figure 3D). Variation in temporal gene expression patterns between rhesus macaques and humans may partly reflect differences in temporal sampling resolution and gene coverage (Figure 3D).

**Figure 3:**
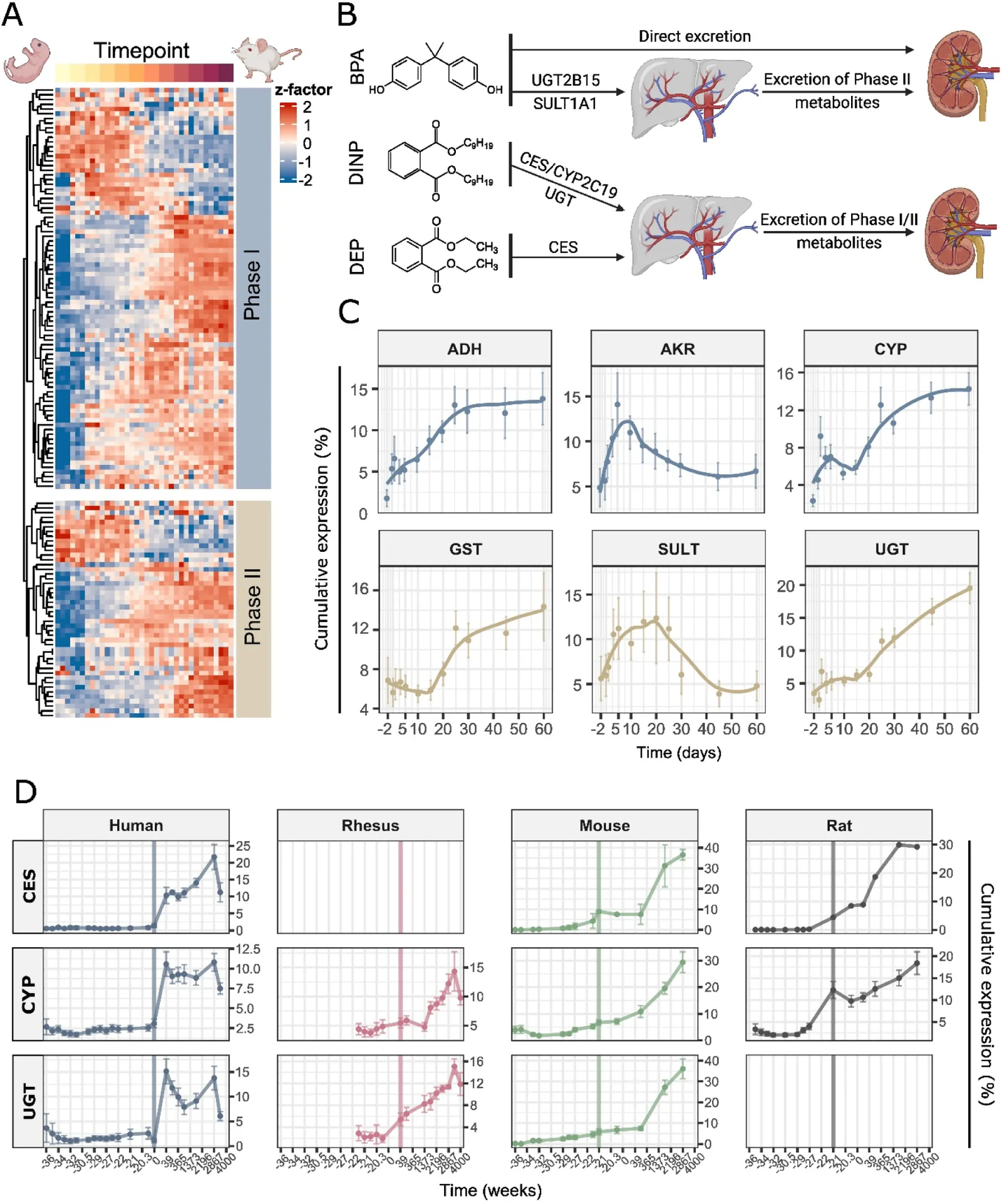
Hepatic expression of genes relevant to Phase I and Phase II biotransformation across maturation of mammalian species (A) Hierarchical clustering of orthologous genes encoding transcripts relevant to Phase I (blue) or Phase II (beige) metabolism in mouse liver across 12 developmental time points, from 2 days before birth to postnatal day 60. Data were retrieved from Gene Expression Omnibus (GSE58827). (B) Schematic overview of xenobiotic metabolism, as exemplified for the contaminants BPA, DINP, and DEP. (C) LOESS-smoothed temporal expression of major Phase I (blue) or Phase II (beige) enzyme families across maturation. For each gene, values were summed across three replicates at each time point and normalized to the cumulative expression of the respective gene across all time points and replicates. Points denote the mean normalized expression across genes within each enzyme family, while error bars represent the corresponding standard error of the mean (SEM). (D) Temporal expression profiles of relevant enzyme families in humans (blue), rhesus macaques (red), mice (green), and rats (dark gray). To account for unequal sample numbers, the expression of each gene was averaged across samples at each time point before normalization to the cumulative mean expression of the gene across time. Normalized gene trajectories were then averaged within each enzyme family. Error bars indicate SEM across genes within each enzyme family. Time axes were standardized to human age (weeks) using the developmental stage alignment proposed by Cardoso-Moreira et al.^20^ The number of experimental replicates may vary across time points and species. In rhesus macaques and rats, no CES- or UGT-family genes, respectively, remained after ortholog mapping and filtering. Birth is indicated by a vertical line. Infographics were created with BioRender.com.

### Pharmacokinetic Model of Parenteral DINP and BPA Exposure in Premature Infants

The experimental data indicate significant BPA and DINP exposure of premature infants via PN. In humans, BPA is rapidly conjugated by UGT2B15 ^35^ and sulfotransferase family 1A member 1 (SULT1A1)^36^ before renal excretion. DINP is thought to undergo hydrolysis to monoisononyl phthalate (MINP) via hepatic CES2 ^37^ followed by Phase I oxidation to 7-OH-MINP, 7-oxo-MINP, and 7-cx-MINP. The oxidation products, in turn, are directly excreted by the kidneys^38,39^ or undergo further glucuronidation prior to excretion.^40^ Alterations in the expression of several involved enzymes were present in human liver samples across maturation (Figure 4A). Consequently, building on our own experiments, one-compartment, first-order, constant-flux pharmacokinetic models were calculated using real-world data from a premature infant treated at our department. Syntheses of the assumptions underlying the models are provided in the Supplementary Document (Chapters S1 and S2). Time-resolved infusion exposure profiles, weight-normalized mean concentrations of xenobiotics as well as corresponding kinetics are displayed in Figure S1.

**Figure 4:**
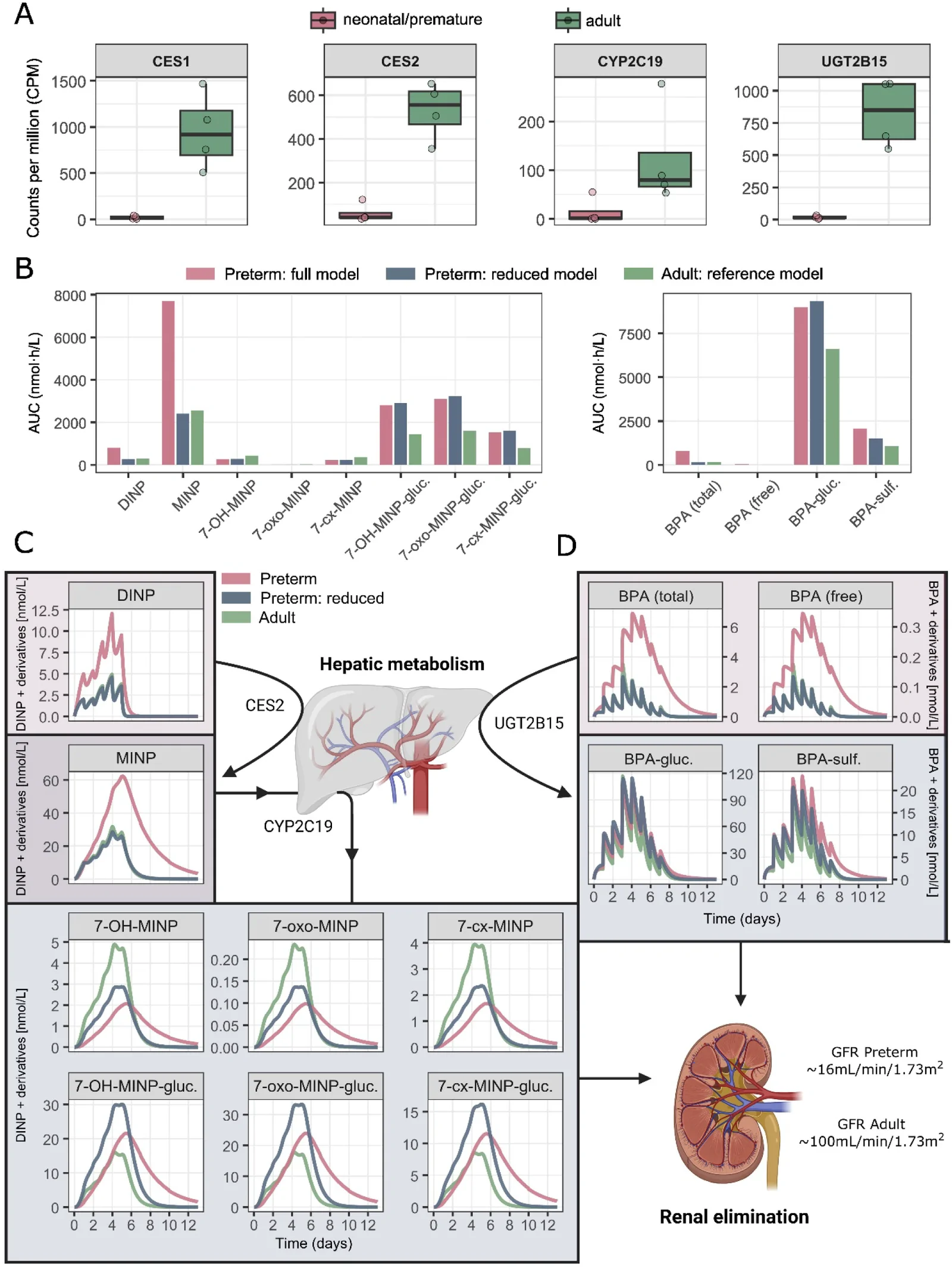
Pharmacokinetic modeling of DINP and BPA exposure using clinical data (A) Hepatic RNA expression of key enzymes relevant to the metabolism of DINP and BPA in human liver tissue of neonates (red; *n* = 4; aggregated from 20 weeks’ gestation to the immediate postnatal period) and adults (green; *n* = 4; 35–55 years). Data were retrieved from Cardoso-Moreira et al.^20^ (B) Predicted cumulative exposure (AUC, nmol·h/L) for DINP, BPA and their respective metabolites. Bar charts compare the preterm full model (red), reduced preterm model (blue) and adult model (green). (C, D) Time-resolved pharmacokinetic modeling of DINP (C) and BPA (D) exposure via parenteral nutrition over the first 14 days after birth. Curves depict the predicted concentrations in the central compartment (nmol/L) for the preterm full model (red), the preterm reduced model (blue) and the adult reference model (green). Metabolism of substances was embedded in a pathway scheme based on relevant literature (Supplementary Document, Chapters S1 and S2). Infographics were created with BioRender.com.

The calculations suggest that DINP is rapidly converted to MINP, with slightly higher concentrations of the parent compound persisting in premature infants due to reduced CES2 expression (Figure 4B). MINP clearance, however, is heavily delayed in premature infants (Figure 4C). Considering maturational changes in CYP2C19 expression, the cumulative exposure of infants to MINP is approximately three times higher than that calculated for adults (Figure 4B). In comparison, systemic exposure to 7-OH-MINP, 7-oxo-MINP, and 7-cx-MINP seems low, suggesting limited toxicological relevance (Figure 4B). For BPA, the data suggest modest exposure of premature infants to the bioactive, nonprotein-bound toxicant (Figure 4B). Even after accounting for developmental differences in hepatic UGT2B15 and SULT1A1 expression, the rapid conjugation of infused BPA dominates over the slow hepatic clearance of systemic nonprotein-bound BPA (Figure 4D). Therefore, although premature infants exhibit a 4.9-fold higher cumulative exposure to total BPA (AUC_(total, full model)_ = 790 nmol·h/L vs AUC_(total, adult)_ = 160 nmol·h/L) and a 4.8-fold higher exposure to free BPA (AUC_(free, full model)_ = 39 nmol·h/L vs AUC_(free, adult)_ = 8.1 nmol·h/L) compared to adults, rapid conjugation and clearance of BPA appear to mitigate overall exposure to the bioactive compound (Figure 4B). However, the potential conversion of free BPA to the highly potent metabolite 4-methyl-2,4-bis­(4-hydroxyphenyl)­pent-1-ene (MBP) upon saturation of UGT- and SULT-mediated Phase II conjugation in preterm infants warrants further attention.

### Exploratory Analysis of Parenteral Nutrition-Associated Exposure in the Plasma of Premature Infants

While the preceding analyses characterize xenobiotic leaching from PN and model systemic distribution, it remains unclear whether PN-associated exposures can be detected in the plasma of premature infants under real-world NICU conditions. Extremely premature infants are exposed to multiple concurrent xenobiotic sources during intensive care, including transfusions, ventilation, and vascular access devices, while blood sampling is limited to residual material from clinically indicated draws. Thus, attributing plasma xenobiotic concentrations, particularly those of substances with short plasma half-lives, to chronic exposure through PN is inherently challenging. However, because HMFA is highly prevalent in heat-sterilized glucose, it may serve as a marker of PN-associated exposure.

To explore this, previously published exposomics data from plasma of premature infants (GA < 28 weeks, birthweight < 1000 g) sampled 28 days postpartum^5^ were accessed and information on PN exposure for the respective patients was retrieved. The clinical characteristics of the included infants are provided in Supplementary Chapter S4. Relevant xenobiotics quantified in both the PN leaching experiments as well as the clinical data set were selected for further analysis (BPA, BBP, NBBS). Mono­(2-ethylhexyl) phthalate (MEHP) was included as a marker of DEHP exposure. When patients were stratified according to PN exposure at the time of blood sampling, HMFA concentrations were significantly higher in infants receiving PN than in infants not receiving PN (*n* = 13 vs *n* = 8, adj. *p*-value = 0.010) (Figure 5A). This effect remained significant after exclusion of infants who received red blood cell transfusions, a second possible source of HMFA, within 3 days prior to blood sampling (adj. *p*-value = 0.036, Chapter S4). Among PN-treated infants (*n* = 13), HMFA concentrations positively correlated with plasma BPA (*r* = 0.67, adj. *p*-value = 0.024), NBBS (*r* = 0.61, adj. *p*-value = 0.035), and MEHP concentrations (*r* = 0.71, adj. *p*-value = 0.024) (Figure 5B). No significant differences in BPA, NBBS, or MEHP levels were observed when directly stratifying infants by the presence of PN (Figure 5C-E, top). Moreover, when infants who were not receiving PN were included in the correlation analyses, associations with exposure compounds were attenuated (Figure 5C-E, bottom). No association between PN and BBP was found, irrespective of sample stratification (Chapter S4). Details on statistical tests as well as unadjusted and adjusted *p*-values for significant and nonsignificant results are provided in the Supplementary Document (Chapter S4).

**Figure 5:**
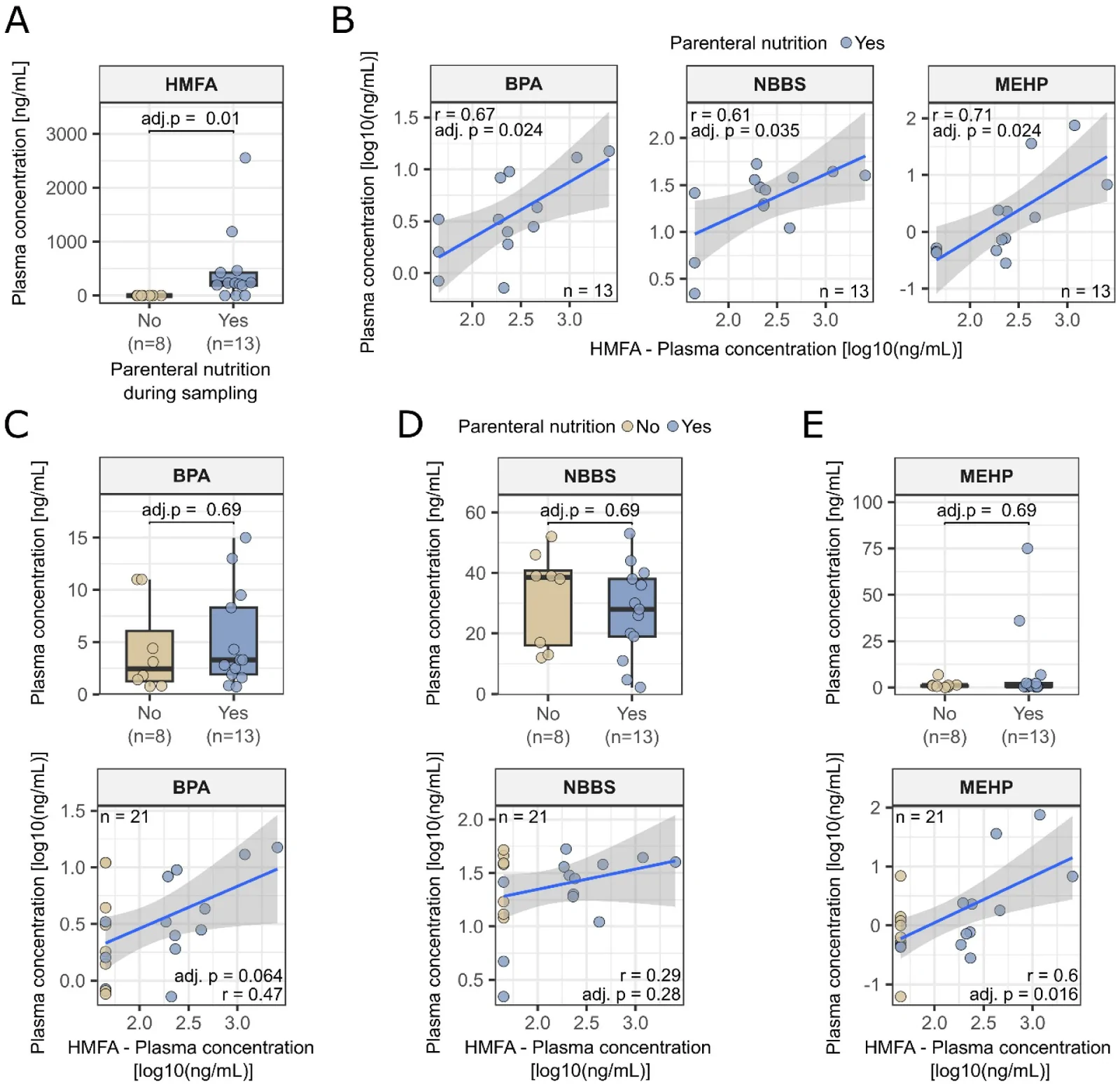
Parenteral nutrition-associated exposure in the NICU-setting (A) HMFA plasma concentrations (ng/mL) in extremely premature infants who received (blue, *n* = 13) or did not receive (beige, *n* = 8) parenteral nutrition at the time of blood sampling. (B) Correlation between HMFA plasma concentration and plasma values of BPA, NBBS, and MEHP in extremely premature infants treated with parenteral nutrition at the time of blood sampling (blue, *n* = 13). (C, D, E) Comparison of plasma concentrations of BPA (top, left), NBBS (top, middle), and MEHP (top, right) between infants who received (blue, *n* = 13) or did not receive (beige, *n* = 8) parenteral nutrition at the time of blood sampling. Correlation between HMFA plasma values and BPA (bottom, left), NBBS (bottom, middle), and MEHP (bottom, right) plasma concentrations in all extremely premature infants included in the cohort (*n* = 21). Points corresponding to infants receiving PN at the time of blood sampling are shown in blue (*n* = 13), whereas those corresponding to infants not receiving PN are shown in beige (*n* = 8). All data included in the panel were reanalyzed from Jamnik et al.^5^

The observed relationship between plasma HMFA values and xenobiotic concentrations in PN-treated infants provides indirect evidence for a contribution of PN to the overall exposure profile of premature infants. The absence of group-specific differences for BPA, NBBS, and MEHP upon stratification by PN alone likely reflects substantial within-group variability arising from concomitant xenobiotic exposures through other interventions associated with a high exposure burden.

## Discussion

Premature infants require intensive medical care, especially those born before 28 weeks of gestation. Interventions including respiratory support,^7^ transfusion,^41^ and PN^10^ often necessitate the use of flexible polyvinyl chloride (PVC), which could introduce potentially toxic xenobiotics into the immature organism. Data from previous studies of our group demonstrated the presence of several plasticizers and industrial additives in the plasma of preterm infants treated in the NICU 28 days postdelivery.^5,42^ Conceptually, exposure from medical interventions can be classified as parenteral or enteral, and as continuous or sporadic. Continuous parenteral exposure may carry a particularly high risk of systemic toxicity, as impaired hepatic biotransformation and renal excretion^43,44^ are likely to potentiate the accumulation and tissue penetration of uncleared lipophilic parent compounds and their derivatives. While intensive care interventions such as hemodiafiltration and extracorporeal membrane oxygenation result in the highest xenobiotic burden,^45^ routine procedures performed in a larger collective of infants, such as respiratory support^46^ and PN^10^ have also been implicated in early life exposure.

Due to the likely diversification of phthalate alternatives in medical products following the European Medical Device Regulation, we investigated the cumulative xenobiotic burden of infant PN in the NICU-setting using a recent exposomic technology covering more than 200 external exposures.^17^ To this end, we simulated the PN regimen of an extremely premature infant born at 28 weeks’ gestation with a birth weight of 1000 g. This scenario was selected instead of a more extreme edge-of-viability model because PN management in such cases is often highly individualized and influenced by clinical factors, including fluid requirements, hemodynamic instability, and center-specific treatment strategies. As a single-center case study, our exposure estimates reflect a relevant clinical reference scenario rather than a generalizable estimate. Nutritional composition may be similar across centers under shared guidelines, but contamination profiles can differ by product brand, compounding practice, and device materials. Complete formulation and leachable data are not routinely available for all products, and a comprehensive assessment would require a large, multinational, multicenter design. Our findings should therefore be interpreted as evidence from one representative exposure pathway rather than a population-wide estimate.

Paralleling reports suggesting increased usage of alternative plasticizers in neonatal PVC-based medicinal products,^3,11,47^ we did not observe DEHP levels above measurement background. Rather, DINP was found to be the most abundant PN contaminant, with a mean daily intake of 19 μg/kg bw/d (AUC_(0–24h)_ = 2.2 × 10^4^ ng·h/mL, Rin = 0.87 mL/h). This is almost four times higher than the value reported by Panneel et al.^10^ Moreover, in contrast to Panneel et al., DINP was not detectable in the aqueous PN samples analyzed in our study. Since DINP is a mixture of phthalic acid isononyl-esters with an octanol–water partition coefficient (log *K*_ow_) of 8.8,^48^ selective partitioning into the lipid phase is plausible. In line with this consideration, the structurally related DEHP (log *K*_ow_ ∼ 7.5)^49^ has previously been shown to accumulate in lipid-based PN.^12,13^ Using hepatic and renal toxicity as critical effects, we derived a provisional prematurity-adjusted parenteral HBGV of 63 μg/kg bw/d for DINP (Table 4). At an EDI of 19 μg/kg bw/d, the calculated prematurity-adjusted HQ was 0.30. Although this value is approximately 1 order of magnitude higher than that reported by Panneel et al.,^10^ DINP exposure via PN remained below the prematurity-adjusted provisional HBGV. Nevertheless, parenteral exposure to doses as low as 48 μg/kg bw/d has been shown to induce changes in the lipid profile of neonatal mice.^50^ Moreover, evidence indicates that DINP and its metabolites interact with peroxisome proliferator-activated receptor alpha (PPARα), an important regulator of hepatic lipid metabolism.^51,52^ These observations are relevant, as other high-molecular-weight phthalates, such as DEHP, have been implicated in the pathogenesis of parenteral nutrition-associated liver disease (PNALD).^13,53,54^ Because PN is administered during periods of high intervention intensity,^1^ effects by sustained DINP administration may be compounded by transient high-level exposures to other long-chain phthalates such as DEHP. Consequently, provisional HBGVs and associated HQs for DINP exposure derived in this study, although adjusted for route of administration and prematurity, may only insufficiently cover possible additive effects of compound mixtures through shared or additive mechanisms of action. Moreover, the preferential leaching of long-chain phthalates, including DINP, into intravenous lipid emulsions^13^ may be of particular relevance, as lipid emulsions themselves have been implicated in the pathogenesis of parenteral nutrition-associated liver disease (PNALD).^12,55^

**Table 4:** Estimated Daily Intake, Provisional HBGVs, and Provisional Hazard Quotients for Xenobiotics Detected in Neonatal Parenteral Nutrition^a^

Compound	Prov. HBGV (μg/kg bw/d)	Preterm-adjusted prov. HBGV (μg/kg bw/d)	PoD source	Critical effect	EDI (μg/kg bw/d)	Prov. hazard quotient (HQ)	Preterm-adjusted prov. HQ
BBP	190	19	EFSA	reproductive toxicity	0.0038	2.0 × 10^–5^	2.0 × 10^–4^
DEP	790	79	EMA	reproductive toxicity	0.053	6.6 × 10^–5^	6.6 × 10^–4^
DINP	150	63	EFSA	hepatic/renal toxicity	19	0.13	0.30
DMP	1.7 × 10^4^	1700	AICIS	maternal toxicity	0.055	3.2 × 10^–6^	3.2 × 10^–5^
MMP	n.a.	n.a.	n.a.	n.a.	0.18	n.a.	n.a.
MnBP	0.22	0.022	ECHA (DBP)	developmental toxicity	0.018	0.081	0.81
MiBP	0.26	0.026	ECHA (DBP read-across)	developmental toxicity	0.018	0.069	0.69
NBBS	100	10	REACH Dossier	hepatic/renal toxicity	0.029	2.9 × 10^–4^	0.0029
BPA	0.04	0.0083	BfR (Liu et al.)	reproductive toxicity	9.5	240	1100
BPA	0.10	0.021	BfR (Srivastava et al.)	reproductive toxicity	9.5	95	450
BPS	200	20	EFSA	immunotoxicity	0.0026	1.3 × 10^–5^	1.3 × 10^–4^
MP	600	60	SCCS	reproductive toxicity	3.8	0.0063	0.063
PP	200	20	EMA	estrogenic activity	0.23	0.0012	0.012
PFBS	83	8.3	EPA	thyroid toxicity	0.0063	7.6 × 10^–5^	7.6 × 10^–4^
HMFA	n.a.	n.a.	n.a.	n.a.	2.2	n.a.	n.a.
Daidzein	10	1.0	SCCS	reproductive toxicity	0.0011	1.1 × 10^–4^	0.0011
Genistein	8.0	0.80	SCCS	estrogenic activity	6.6 × 10^–4^	8.3 × 10^–5^	8.3 × 10^–4^
MPCA	n.a.	n.a.	n.a.	n.a.	0.019	n.a.	n.a.

a AICIS = Australian Industrial Chemicals Introduction Scheme; BfR = German Federal Institute for Risk Assessment; ECHA = European Chemicals Agency; EDI = Estimated daily intake; EFSA = European Food Safety Authority; EMA = European Medicines Agency; EPA = U.S. Environmental Protection Agency; HBGV = Health-Based Guidance Value; HQ = Hazard quotient, n.a. = not applicable; PoD = Toxicological point of departure; REACH = Registration, Evaluation, Authorization and Restriction of Chemicals; SCCS = Scientific Committee on Consumer Safety. Due to their low detection frequency, BPE and PFHxA were excluded from the analysis. The detailed search strategy, assumptions underlying respective calculations and sources are summarized in the Supplementary Document (Chapter S3). Importantly, while the calculated HBGVs are based on evidence reviewed by governmental and regulatory bodies they do not reflect official decisions by these authorities and should therefore be regarded as provisional values. HQs were calculated using unrounded EDI and HBGV values. Underlying EDI calculations were performed using rounded values displayed in Table 3. Scientific notation was used for values <10^–3^ or >10^4^. Values were rounded to two significant digits.

The estimated daily intake values for other phthalates, including DEP (DF_(aq/lip)_ = 100%/100%, EDI_(mean)_ = 0.053 μg/kg bw/d), DMP (DF_(aq/lip)_ = 100%/0%, EDI_(mean)_ = 0.055 μg/kg bw/d), BBP (DF_(aq/lip)_ = 0%/75%, EDI_(mean)_ = 0.0038 μg/kg bw/d), and MnBP/MiBP (DF_(aq/lip)_ = 94%/0%, EDI_(mean)_ = 0.018 μg/kg bw/d) were three to 4 orders of magnitude lower than that of DINP. In contrast, HMFA, a product of the Maillard reaction during heat sterilization, was present in PN at comparatively high concentrations (DF_(aq/lip)_ = 100%/100%, EDI_(mean)_ = 2.2 μg/kg bw/d). Previously, we also detected HMFA in the micromolar range in plasma samples of extremely premature infants.^5^ Since HMFA predominantly derives from glucose degradation, it may represent a candidate marker of PN exposure. Indeed, HMFA plasma concentrations were tightly correlated with leaching-associated exposures in our exploratory analysis of PN-treated infants (Figure 5). MP was also present in PN at comparatively high concentrations (DF_(aq/lip)_ = 94%/75%, EDI_(mean)_ = 3.8 μg/kg bw/d). In addition to being common background contaminants in exposure analyses due to their ubiquitous use in personal care products,^56^ parabens are also added to parenteral pharmaceuticals as antimicrobial preservatives,^57^ possibly explaining their presence in PN. Although none of these compounds exceeded their provisional parenteral HBGV (Table 4), the calculated prematurity-adjusted HQs for MnBP (0.81) and MiBP (0.69) approached 1. While our estimates do not reflect official governmental body decisions and are likely conservative (Chapter S3), the shared endocrine mode of action with other phthalates, including DEHP and BBP, as recognized by EFSA,^58^ warrants further attention.

BPA was the only compound present in PN for which the estimated daily intake exceeded all guideline-derived and calculated provisional prematurity-adjusted HBGVs. At an EDI of 9.5 μg/kg bw/d, estimated HQs ranged across 4 orders of magnitude. Even the most permissive benchmark available, an adult chronic oral permitted daily exposure of 8.3 μg/kg bw/d proposed by the EMA in the draft ICH Q3E Guideline for extractables and leachables, yielded an HQ of 1.1.^33^ By comparison, the adult chronic parenteral HBGV of 0.083 μg/kg bw/d estimated in the same document resulted in an HQ of 114. The oral adult HBGV calculated by the BfR in 2023 yielded an HQ of 48 ^59^ whereas the rather permissive 2015 EFSA TDI of 4 μg/kg bw/d resulted in an HQ of 2.4.^60^ Given the draft status of EMA-proposed HBGVs for BPA,^33^ we additionally calculated parenteral, prematurity-adjusted HBGVs using PoDs proposed by the BfR.^59^ We selected these PoDs, as their underlying apical end points more closely align with the EMA framework than the immunotoxicity end points referred to by the EFSA (TDI = 2 × 10^–4^ μg/kg bw/d; HQ = 4.8 × 10^4^).^61^ The resulting calculated provisional parenteral HBGVs of 0.10 and 0.040 μg/kg bw/d yielded HQs of 95 and 240, values that rose to 450 and 1100 after adjustment for prematurity. Pharmacodynamic studies on BPA have suggested effects on human T-cells^62^ and antigen-presenting cells^63,64^ at mid- to high-nanomolar concentrations. Interactions with endocrine receptors such as human estrogen receptor alpha (ERα) and androgen receptor (AR) have been reported at an EC50_(ERα)_ of 270 nmol/L and IC50_(AR)_ of 2300 nmol/L, respectively.^65^ In addition, effects on insulin secretion in isolated rat pancreatic cells have been observed at concentrations between 10 and 50 nmol/L.^66,67^ The predicted peak concentration of free BPA in our prematurity-adjusted model (0.35 nmol/L) lies approximately 30-fold below the lowest of these reported concentrations and nearly three and 4 orders of magnitude below the reported ERα and AR potencies. However, the potentially increased fraction of BPA shunted toward oxidative metabolism, owing to insufficient UGT2B15 ^28^ and SULT1A1 ^29^ expression, and the consequent formation of the approximately 160-fold more potent metabolite MBP (EC50_(ERα)_ ∼ 1.7 nmol/L, EC50_(ERβ)_ ∼ 2.2 nmol/L),^68^ warrant further attention.

Regarding PFBS, no defined health-based guidance value exists within the EU. The only European anchor, a tolerable weekly intake of 0.0044 μg/kg bw/week proposed by EFSA, covers the summed exposure to PFOA, PFNA, PFHxS, and PFOS.^69^ When directly applied to the EDI of PFBS (0.0063 μg/kg bw/d), it yields an HQ of 10. However, this likely overstates the hazard of PFBS, as plasma clearance in humans is high (*t*_1/2_ ∼ 50 d)^70^ compared to other PFAS.^70,71^ Moreover, the PN-attributable intake of PFBS remains more than 3-fold below the cumulative daily oral intake of PFAS by breastfed infants in Europe (0.020 μg/kg bw/d), a route with near-complete absorption.^70^ Outside the EU, the US EPA has derived a chronic reference dose of 0.3 μg/kg bw/d from PFBS-induced effects on thyroid function and development in gestationally exposed mice,^72^ corresponding to an HQ of 0.021. Recalculating from the same PoD using an EMA-informed framework, we obtained a provisional parenteral, prematurity-adjusted HBGV of 8.3 μg/kg bw/d (HQ = 7.6 × 10^–4^) for PFBS. Thus, PFBS exposure through PN is likely not of concern.

In summary, the most abundant contaminants in simulated neonatal PN were DINP, BPA, MP, and HMFA (EDIs: 19, 9.5, 3.8, and 2.2 μg/kg bw/d), of which only BPA exceeded calculated provisional and official HBGVs. Partitioning of xenobiotics into the lipid and aqueous mixtures broadly tracked reported log Kow values at the extremes (e.g., DINP, HMFA). Prediction was less reliable for analytes in the intermediate range (e.g., BPA), likely reflecting sufficient absolute aqueous solubility of these compounds and their independent presence in the solutions or the devices used to administer them. In the plasma of extremely premature infants treated with PN, concentrations of HMFA, a contaminant of heat-sterilized glucose and potential marker of PN exposure, covaried with BPA, NBBS, and MEHP. However, apart from HMFA, direct comparison of infants with and without PN revealed no significant group-wise differences in xenobiotic exposure (Figure 5). Studies have repeatedly identified interventions such as erythrocyte transfusion and (non)­invasive ventilation as considerable exposure sources in the NICU-setting.^3,47,73^ As analytical measurements in the reanalyzed study were ethically restricted to residual material from clinically indicated blood draws, sampling could not be coupled to intervention-free windows. Thus, exposure from ventilation, transfusion, or pharmacotherapy via (in)­direct contact with BPA, NBBS, or DEHP-containing materials or solutions may have increased between-subject variance in plasma concentrations and impeded the detection of PN-associated effects in group-wise comparisons. The inability of the PN-associated effect to overcome this variability, despite being detectable through correlation with HMFA, suggests that PN adds to, but does not dominate, the xenobiotic exposure profile of premature infants. However, given the single sampling time point at 28 days postpartum, the limited sample size, the sporadic timing of sampling, the close relationship between disease severity and intervention frequency, and the incomplete information on the magnitude of concomitant xenobiotic exposures, this interpretation warrants further prospective investigation under controlled conditions. Nevertheless, because BPA intake exceeded all identified and calculated HBGVs and structurally related phthalates may exert additive toxicological effects, eliminating BPA-releasing components from PN administration sets and reducing the cumulative phthalate burden represent actionable targets for risk reduction.

Future studies should focus on other common sources of long-term parenteral exposure with high cumulative toxicological burden, such as mechanical ventilation. Moreover, competition for shared detoxification pathways should be taken into account,^74^ especially since concurrent exposure of infants to pharmaceuticals at substantially higher concentrations may further delay xenobiotic clearance. Additionally, substances with shared mechanisms of action should be evaluated using mixture assessment factors to account for additive toxicological effects. Finally, potentially elevated levels of the highly bioactive metabolite MBP, possibly related to insufficient hepatic conjugation in premature infants, warrant further investigation.

## Limitations

This study has several methodological limitations. Leaching from individual devices or components in isolation was not investigated. Consequently, specific xenobiotics cannot be attributed to particular materials or connectors within the infusion pathway. The simulation reflected only one PN regimen administered at a fixed infusion rate. Moreover, while the simulated PN-mixtures align with current ESPGHAN guidelines, the representative nutritional composition does not imply universal representativeness of chemical contamination profiles. Since flow conditions can influence compound mass transfer, varying infusion rates could alter compound migration into the infused solutions. From an analytical perspective, nontargeted analysis/screening^75–77^ might allow for identification of exposures not covered by the targeted approach. For simplicity, altered migration was not factored into the pharmacokinetic model. Moreover, the pharmacokinetic model is parametrized on the clinical course of one infant. Thus, interindividual variability in potential nutritional regimens, flow rates, and growth is not captured and the predicted exposures should be read as a representative scenario rather than a population estimate. Individual changes in renal clearance based on clinical GFR estimates were not modeled. Plasma concentrations of DINP and several other contaminants identified in parenteral nutrition were not available in the data set reported by Jamnik et al.^5^ and were therefore not included in the analysis. Because the original measurements were performed using a low-resolution targeted mass spectrometry method, post hoc reanalysis of the samples for these analytes was not possible. Additionally, the plasma analysis is exploratory and not intended to validate the pharmacokinetic model. Validation would require serial sampling across defined intervention-free windows, which is not feasible, as sampling is restricted to residual material from clinically indicated blood draws. While calculated HBGVs are based on evidence reviewed by governmental and regulatory bodies, they do not reflect official decisions by these authorities and should be interpreted with caution. The use of an FQPA-informed sensitivity factor of 10 to adjust for prematurity constitutes a pragmatic and conservative choice for risk assessment. Additional limitations of the HBGV approach are summarized in detail in Supplementary Chapter S3. Finally, plasticizer release was not modeled under incubator temperature conditions.

## Supplementary Material





## Data Availability

Raw LC-MS data have been uploaded to the MetaboLights repository (accession number: REQ20260318217919). Summarized information on the experimental runs, including batch information for the pharmaceuticals and consumables used for sample preparation, as well as details of the analytical measurements, is provided in the Supplementary Tables (Tables S1–S6). The code for the xenobiotic analyses and the pharmacokinetic model can be accessed via the link https://github.com/wisgrill-lab/Parenteral-Nutrition-as-a-Source-of-Xenobiotic-Exposure-in-Preterm-Infants. Additional information on modeling decisions, clinical characteristics, statistical analyses, as well as the methodology underlying the identification of suitable HBGVs is provided in the Supplementary Document (Chapters S1–S4).

## Notes

Supplier information of the chemicals used in analytical experiments (Table S1); Supplier and batch information for pharmaceuticals and consumables used in the experimental runs (Table S2); Concentration of the analytes in the solvent calibration and in the spiked matrix standards (Table S3); LC-Gradient (Table S4); MRM transitions (Table S5); Solvent calibration curve range and linearity (coefficient of determination, *R* ^2^), process-blank correction status, signal suppression/enhancement (SSE), limit of detection (LOD), and limit of quantification (LOQ) for plastic-related chemicals and other xenobiotics detected in the parenteral nutrition solution and the vitamin solution (Table S6) (XLSX)
Weight-based values from pharmacokinetic modeling of DINP and BPA exposure (Figure S1); Theoretical assumptions underlying the DINP pharmacokinetic model (Chapter S1); Theoretical assumptions underlying the BPA pharmacokinetic model (Chapter S2); Additional information on the data included in Table 4 (Chapter S3); Clinical characteristics of the study cohort and statistical comparisons (Chapter S4) (PDF)
