# Supplementary material for: Parenteral Nutrition as a Source of Xenobiotic Exposure in Preterm Infants in a Single-Center Case Study
Source: Environ Sci Technol. 2026 Oct 19;60(43):30512–29. doi: 10.1021/acs.est.6c12407 (PMC13738794; doi:10.1021/acs.est.6c12407)
Supplement: Supplementary file 1 [file es6c12407_si_002.pdf]

Parenteral Nutrition as a Source of Xenobiotic Exposure in Preterm Infants in a Single-Center Case Study

Michael Eigenschink [1,2], Nika Wattaul [1,2], Sabrina Berger [3], Jesse Peach [3], Ernst W. Müllner [4], Angelika Berger [1,2], Benedikt Warth [3,5], Harald H. Sitte [6,7,8], Lukas Wisgrill [1,2,5*]

[1] Department of Pediatrics, Division of Neonatology, Pediatric Intensive Care and Neuropediatrics, Medical University of Vienna, Waehringer Guertel 18-20, 1090 Vienna, Austria

[2] Comprehensive Center for Pediatrics, Medical University of Vienna, Waehringer Guertel 18-20, 1090 Vienna, Austria

[3] Department of Food Chemistry and Toxicology, University of Vienna, Waehringer Strasse 38, 1090 Vienna, Austria

[4] Center for Medical Biochemistry, Max Perutz Labs, Medical University of Vienna, Dr.-Bohr-Gasse 9, 1030 Vienna, Austria

[5] Exposome Austria, National Research Infrastructure and EIRENE-AT Node, 1090 Vienna, Austria

[6] Center for Physiology and Pharmacology, Institute of Pharmacology, Medical University of Vienna, Waehringer Strasse 13a, 1090 Vienna, Austria

[7] Hourani Center for Applied Scientific Research, Al-Ahliyya Amman University, 19328 Amman, Jordan

[8] AddRess Center for Addiction Research and Science, Medical University of Vienna, Waehringer Strasse 13a, 1090 Vienna, Austria

* Corresponding author
Email: lukas.wisgrill@meduniwien.ac.at
Telephone: +43 1 40400 29300
Fax: +43 1 40400 29290

**TABLE OF CONTENTS**		**Page**
**Figure S1.**	Weight-based values from pharmacokinetic modeling of DINP and BPA exposure	2-3
**Chapter S1**.	Theoretical assumptions underlying the DINP pharmacokinetic model	4-9
**Chapter S2**.	Theoretical assumptions underlying the BPA pharmacokinetic model	10-14
**Chapter S3**.	Additional information on the data included in Table 4	15-29
**Chapter S4.**	Clinical characteristics of the study cohort and statistical comparisons	30-31

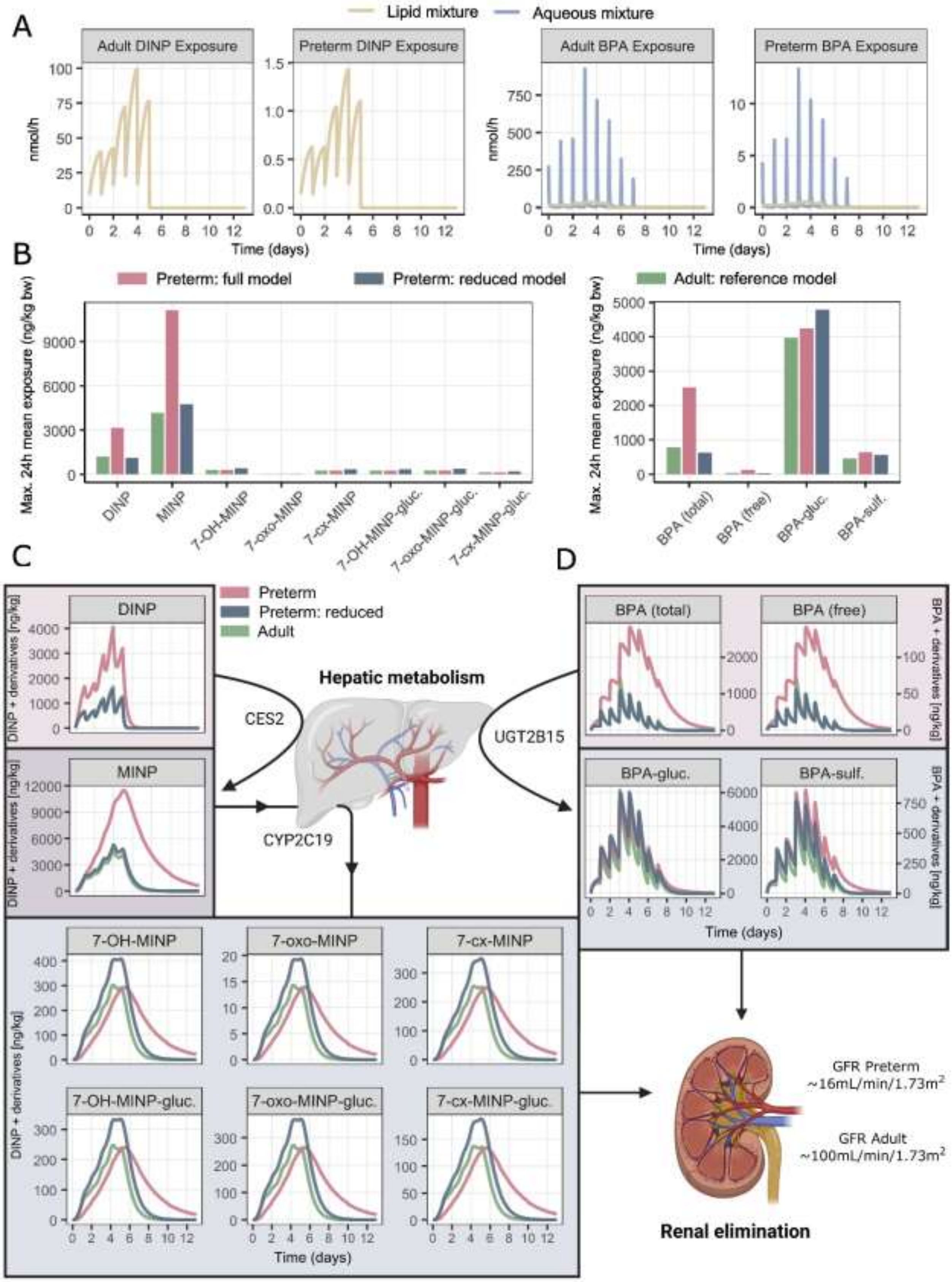

Figure S1. Weight-based values from pharmacokinetic modeling of DINP and BPA exposure

(A) DINP (left) and BPA (right) xenobiotic influx rates (nmol/h) calculated from our kinetic data for aqueous (blue) and lipid (beige) samples. Values served as input for subsequent pharmacokinetic calculations. (B) Highest

average exposure within a consecutive 24 h timeframe (ng/kg) for DINP, BPA and their metabolites. Bar charts: preterm full model (red), reduced preterm model (blue), adult model (green). (C, D) Time-resolved pharmacokinetic modeling of DINP (C) and BPA (D) exposure via parenteral nutrition over the first 14 days after birth. Curves depict exposures (ng/kg) for the preterm full model (red), the preterm reduced model (blue) and the adult reference model (green). Metabolism of substances was embedded in a pathway scheme based on relevant literature (supplementary document, Chapter S1 & S2). Infographics were created with BioRender.com.

Chapter S1 - DINP model

Data on the metabolization and excretion of diisononyl phthalate (DINP) in humans are limited. Most studies focus on biomonitoring (1–3), with only two oral dosage studies tracing DINP, conducted by Koch et al. (4) and Anderson et al. (5), respectively.

These two publications suggest that only between 2% (4) and 3% (5) of the orally applied DINP dose can be detected as monoisononyl phthalate (MINP) in urine within 48 h. However, 20.2% of the applied dose was recovered as mono-hydroxy-isononyl phthalate (OH-MINP), 10.6% as mono-oxo-isononyl phthalate (oxo-MINP), and 10.7% as mono-carboxy-isononyl phthalate (cx-MINP) during the same timeframe (total recovered dose = 43.6%) (4). Anderson et al. reported a similar pattern, albeit with a slightly lower total dose recovery of 32.9% (5).

Theoretical assumptions of DINP to MINP conversion

Since only 43.6% of substance was recovered in urine within 48h (4), the fate of the remaining 56.4% must be addressed. A common explanation, especially for oral dosing, is the partitioning of the substance into feces due to insufficient enteral absorption. Unfortunately, for DINP, no human plasma data for bioavailability after oral dosing exist. However, two studies in rats investigated plasma levels and fecal excretion of DINP after oral administration (6,7). F-344 rats administered 500 mg/kg (^{14}C)-spiked DINP orally excreted 32.8% of the dose in feces within 24 h with 67% of the recovered fecal substance being unchanged parent DINP (6). In contrast, Sprague-Dawley rats receiving 100 mg/kg DINP orally excreted 13.6% of the administered dose as unchanged DINP in feces within 24h to 48h. However, when DINP was administered intravenously at 10 mg/kg, no measurable excretion of parent DINP in feces was observed (7). Additionally, Iwata et al. observed lower levels of fecal excretion in mice with humanized livers (7.7%) compared to control mice (37.3%) within 48h after oral administration of 50 mg/kg (^{14}C)-spiked DINP. In these mice, only 7.6% (control) and 0.2% (humanized liver) of the fecal DINP corresponded to the unchanged parent compound (8).

Considering these animal data, it seems plausible that only a minor fraction of orally administered DINP is not bioavailable. We computed this fraction from animal data as 10.8% (calculated as [(32.8 * 0.67) + 13.6 + 7.6 + 0.2] / 4 = 10.8%). This value was used to correct the calculation of MINP metabolite fractionation, which is based on human urinary data (4). Given the rapid metabolization of DINP to MINP and the reduced fecal excretion of DINP in humanized liver mice, it is plausible that, after intravenous administration, conversion of DINP to MINP, MINP oxidation and the metabolization of oxidized derivatives of MINP into a diverse set of unknown (and thus unquantified) metabolites might account for the fraction of applied substance not detected in human urine (4). Since no evidence was found for direct renal elimination of DINP in humans, $CL_{(DINP)}$ was assumed to be equal to $CL_{(DINP,\ hepatic)}$.

Approximation of volume of distribution and clearance of DINP

Calculation of DINP concentrations warrants knowledge of plasma $V_{d(DINP)}$. Since no data on the V_d of DINP are available for humans, we retrieved $D_{(DINP)}$ and $C_{0(DINP)}$ from an intravenous dosage study in rats and calculated $V_{d(DINP)}$ as $V_{d(DINP)} = D_{(DINP)} / C_{0(DINP)}$ (7). Next, we scaled $CL_{(DINP, rat)}$ to $CL_{(Adult)}$ using a published scaling factor of $CL_{(human, adult)} = 0.152 * CL_{(rat)}$ (9). Assuming that $CL_{(DINP)} = CL_{(DINP, hepatic)}$, we multiplied this value by the hepatic volume ratio (0.016) between adults (mean = 1360 cm^3) (10) and preterm infants (median = 21.1 cm^3) (11). Since reliable experimental data on hepatic blood flow in preterm infants are unavailable, we could not account for possible differences in hemodynamics that might influence $CL_{(DINP, hepatic, preterm)}$.

Studies indicate that the hydrolysis of high-molecular-weight-phthalates like DEHP and DINP to their monoesters (MEHP, MINP) after parenteral administration is primarily driven by hepatic carboxylesterase 2 (CES2) (12). Consequently, we further adapted the volume-scaled $CL_{(DINP, hepatic, preterm)}$ by a maturation factor based on the ratio of CES2 protein expression (0.34) measured in neonatal (59.8 pmol/mg) and adult (174.1 pmol/mg) hepatic microsomes (13).

Assumptions for MINP clearance calculation

As previously mentioned, it is plausible that most intravenously administered DINP is converted to MINP. Since renal excretion of MINP in humans is negligible (4), $CL_{(MINP)}$ was assumed to equal $CL_{(MINP, hepatic)}$. Unfortunately, plasma clearance of MINP has not been determined for humans. However, a recent publication calculated MINP plasma clearance from oral DINP administration in humanized-liver mice and scaled values to that of humans (CL = 2.23 L/h) (14). We used this published value for subsequent calculations by further scaling it according to the hepatic volume ratio (0.016) between adults and preterm infants and multiplying it with the ratio of hepatic microsomal cytochrome P450 family 2 subfamily C member 19 (CYP2C19) protein content (0.30) between neonates (4.5 pmol/mg, calculated as (3.4 + 5.6) / 2 = 4.5 pmol/mg) and adults (14.9 pmol/mg) (15), as CYP2C19 polymorphisms have been shown to quantitatively alter DINP metabolite concentrations in humans (16).

For calculating MINP concentrations in the systemic compartment, no V_d could be derived from the literature. However, experimental evidence suggests a V_d of 0.8 for the lipophilic parent compound DINP (7). Since we use the extracellular fluid volume of a preterm infant (V_d = 0.46 L/kg) (17) as a proxy for the volume of distribution of polar Phase I oxidative metabolites of MINP (calculations introduced below), we selected V_d for MINP ($V_{d(MINP)}$ = 0.63 L/kg) to fall between the extracellular fluid volume (oxidative metabolites, V_d = 0.46 L/kg) and the calculated V_d of the parent diester DINP ($V_{d(DINP)}$ = 0.8 L/kg).

Fractionation of MINP metabolites

Based on published human data, Phase I metabolites constitute 41.5% of the urinary excreted oral DINP dose (4). Assuming the previously calculated 10.8% loss due to incomplete parent compound absorption in the intestine, 53.5% of the absorbed oral dose remains unaccounted for in the urinary

quantifications (calculated as 100 – [100 / (100 – 10.8)] * 41.5 = 53.5%). Because Phase I oxidation of MINP is expected to result in a greater diversity of metabolites compared to the conversion of parent compound to MINP, we partitioned $CL_{(MINP)}$ into $CL_{(MINP, unknown)} = CL_{(MINP)} * 0.535$ and $CL_{(MINP, known)} = CL_{(MINP)} * 0.465$. For the 46.5% known Phase I metabolites we then calculated MINP metabolite-specific clearance based on fractional excretion values observed in human urine as $CL_{(OH-MINP)} = CL_{(MINP, known)} * (0.202 * [100 / 41.5])$; $CL_{(oxo-MINP)} = CL_{(MINP, known)} * (0.106 * [100 / 41.5])$, and $CL_{(cx-MINP)} = CL_{(MINP, known)} * (0.107 * [100/41.5])$ (4).

Approximation of MINP-metabolite clearances

Since plasma half-lives ($t_{1/2}$) for MINP-metabolites are unknown in humans and animals, we assumed that the urinary $t_{1/2}$ of OH-MINP, oxo-MINP, and cx-MINP reflect plasma $t_{1/2}$ under constant renal filtration without active tubular secretion and reabsorption. We therefore used $t_{1/2}$ values between 4h and 8h, as published by Anderson et al., for subsequent calculations of clearance (5). Specifically, we adopted a $t_{1/2}$ of 6h for OH-, and oxo-MINP and 8h for cx-MINP, as the latter generally showed a slower clearance (5). We then scaled the calculated renal clearances by multiplication with a ratio (0.010) corresponding to differences in body surface area (BSA) adjusted glomerular filtration rates (GFR) between premature infants born at 28 weeks of gestation (GFR = 16.2 mL/min/m^2, BSA = 0.109 m^2) (18,19) and adults (GFR = 101 mL/min/m^2, BSA = 1.73 m^2) (20). We specifically used reference values for healthy adults aged 35-40 years that correspond to the study population of Anderson et al. from which we obtained $t_{1/2}$ values (mean age = 35.6 years) (5).

In our setting, the calculation of clearances requires knowledge of V_d values for each compound. Unfortunately, V_d values for OH-, oxo-, and cx-MINP, as well as their chemical proxies mono(2-ethyl-5-hydroxyhexyl) phthalate (5-OH-MEHP), mono(2-ethyl-5-oxohexyl) phthalate (5-oxo-MEHP), and mono(2-ethyl-5-carboxypentyl) phthalate (5-cx-MEHP) are unknown. Assuming that the oxidation products of Phase I metabolism are more polar than the diester DINP and the hydrolyzed monoester MINP, we selected $V_{d(Phase\ I)}$ to correspond to the extracellular volume (ECV). Since the ECV changes during maturation, we derived respective values for our setting from published data on sucrose distribution in preterm infants (17).

OH-MINP, oxo-MINP and cx-MINP have been shown to be approximately 45%, 95% and 35% glucuronidated (1). Since urinary $t_{1/2}$ values for respective compounds were measured after enzymatic deconjugation of glucuronic acid, calculated clearances constitute an aggregate value for both unconjugated and conjugated fractions (5). Unfortunately, neither kinetics for Uridine 5′-diphospho-glucuronosyltransferase (UGT)-mediated reactions, nor in vivo half-life times of Phase II metabolites have been reported, effectively preventing us from mathematically modeling conversion and clearance. Nevertheless, we approximated abundances of Phase II metabolites by multiplying the derived amounts of OH-MINP (* 0.45), oxo-MINP (* 0.95) and cx-MINP (* 0.35) with the respective fractions of glucuronidated compounds detected in urine. We then calculated

concentrations of these polar Phase II metabolites by assuming their volume of distribution to approximate that of plasma. For premature infants, this volume is calculated as $V_{d(Plasma)}$ = 0.036 L/kg, using 70 ml/kg as a reference for preterm blood volume (21) and assuming a hematocrit of 48.9% (28 weeks of gestational age) (22).

Compilation of reduced models

To better understand possible differences in DINP pharmacokinetics in relation to prematurity, two reduced models were calculated. The first includes all aspects of the "Preterm: full model" but excludes the additional scaling of DINP- and MINP clearance by maturational differences in CES2 and CYP2C19 expression. This allows the visualization of potential enzyme maturation-dependent effects on DINP elimination. The second model was designed to compare DINP pharmacokinetics in preterm infants to a 70 kg reference adult. In this model, the infusion rate was adjusted using the formula $Vin_{(adult,\ i)} = ([1 / BW_{(day,\ i)}] * Vin_{(preterm,\ i)}) * 70$, to emulate equal daily exposure. Furthermore, factors used to scale hepatic clearance (e.g., liver volume and protein expression), as well as factors used to adapt renal clearance were set to 1. Volumes of distribution for the extracellular volume and plasma were set to their adult counterparts of $V_{d(ECV,\ adult)}$ = 0.2 L/kg and $V_{d(Plasma,\ adult)}$ = 0.05 L/kg, respectively (23). Consequently, $V_{d(MINP,\ adult)}$ was set to 0.5 L/kg. Since adult clearances for a reference 70 kg human were used to inform our preterm infant calculations, omitting the scaling factors, changing volume parameters and adjusting exposure rates yielded an adult reference model suitable for comparison.

List of pharmacokinetic abbreviations and their respective units

CL	Clearance	L/h
V_d	Volume of distribution	L/kg or L, depending on calculation
$t_{1/2}$	Half-life	hours
Vin	Infusion rate	mL/h
BW	Body weight	kg

References

1. Silva MJ, Reidy JA, Preau JL, Needham LL, Calafat AM. Oxidative metabolites of diisononyl phthalate as biomarkers for human exposure assessment. Environ Health Perspect. 2006 Aug;114(8):1158–61. doi:10.1289/ehp.8865 PubMed PMID: 16882519; PubMed Central PMCID: PMC1552017.

2. Trasande L, Nelson ME, Alshawabkeh A, Barrett ES, Buckley JP, Dabelea D, et al. Prenatal phthalate exposure and adverse birth outcomes in the USA: a prospective analysis of births and estimates of attributable burden and costs. The Lancet Planetary Health. 2024 Feb;8(2):e74–85. doi:10.1016/S2542-5196(23)00270-X

3. Lee I, Pälmke C, Ringbeck B, Ihn Y, Gotthardt A, Lee G, et al. Urinary Concentrations of Major Phthalate and Alternative Plasticizer Metabolites in Children of Thailand, Indonesia, and Saudi

Arabia, and Associated Risks. Environ Sci Technol. 2021 Dec 21;55(24):16526–37. doi:10.1021/acs.est.1c04716

4. Koch HM, Angerer J. Di-iso-nonylphthalate (DINP) metabolites in human urine after a single oral dose of deuterium-labelled DINP. International Journal of Hygiene and Environmental Health. 2007 Feb;210(1):9–19. doi:10.1016/j.ijheh.2006.11.008

5. Anderson WAC, Castle L, Hird S, Jeffery J, Scotter MJ. A twenty-volunteer study using deuterium labelling to determine the kinetics and fractional excretion of primary and secondary urinary metabolites of di-2-ethylhexylphthalate and di-iso-nonylphthalate. Food and Chemical Toxicology. 2011 Sep;49(9):2022–9. doi:10.1016/j.fct.2011.05.013

6. McKee RH, El-Hawari M, Stoltz M, Pallas F, Lington AW. Absorption, disposition and metabolism of di-isononyl phthalate (DINP) in F-344 rats. J of Applied Toxicology. 2002 Sep;22(5):293–302. doi:10.1002/jat.861

7. Hou ML, Chang LW, Chiang CJ, Tsuang YH, Lin CH, Tsai TH. Pharmacokinetics of di-isononyl phthalate in freely moving rats by UPLC–MS/MS. International Journal of Pharmaceutics. 2013 Jun;450(1–2):36–43. doi:10.1016/j.ijpharm.2013.04.033

8. Iwata H, Goto M, Sakai N, Suemizu H, Yamazaki H. Predictability of human pharmacokinetics of diisononyl phthalate (DINP) using chimeric mice with humanized liver. Xenobiotica. 2019 Nov 2;49(11):1311–22. doi:10.1080/00498254.2018.1564087

9. Tang H, Hussain A, Leal M, Mayersohn M, Fluhler E. Interspecies Prediction of Human Drug Clearance Based on Scaling Data from One or Two Animal Species. Drug Metabolism and Disposition. 2007 Oct;35(10):1886–93. doi:10.1124/dmd.107.016188

10. Kromrey ML, Ittermann T, vWahsen C, Plodeck V, Seppelt D, Hoffmann RT, et al. Reference values of liver volume in Caucasian population and factors influencing liver size. European Journal of Radiology. 2018 Sep;106:32–7. doi:10.1016/j.ejrad.2018.07.005

11. Szpinda M, Paruszewska-Achtel M, Woźniak A, Mila-Kierzenkowska C, Elminowska-Wenda G, Dombek M, et al. Volumetric Growth of the Liver in the Human Fetus: An Anatomical, Hydrostatic, and Statistical Study. BioMed Research International. 2015;2015:1–8. doi:10.1155/2015/858162

12. Isobe T, Ohkawara S, Mori Y, Jinno H, Tanaka-Kagawa T, Hanioka N. Hydrolysis of dibutyl phthalate and di(2-ethylhexyl) phthalate in human liver, small intestine, kidney, and lung: An in vitro analysis using organ subcellular fractions and recombinant carboxylesterases. Chemico-Biological Interactions. 2023 Feb;372:110353. doi:10.1016/j.cbi.2023.110353

13. Boberg M, Vrana M, Mehrotra A, Pearce RE, Gaedigk A, Bhatt DK, et al. Age-Dependent Absolute Abundance of Hepatic Carboxylesterases (CES1 and CES2) by LC-MS/MS Proteomics: Application to PBPK Modeling of Oseltamivir In Vivo Pharmacokinetics in Infants. Drug Metabolism and Disposition. 2017 Feb;45(2):216–23. doi:10.1124/dmd.116.072652

14. Miura T, Suemizu H, Goto M, Sakai N, Iwata H, Shimizu M, et al. Human urinary concentrations of monoisononyl phthalate estimated using physiologically based pharmacokinetic modeling and experimental pharmacokinetics in humanized-liver mice orally administered with diisononyl phthalate. Xenobiotica. 2019 May 4;49(5):513–20. doi:10.1080/00498254.2018.1471753

15. Koukouritaki SB, Manro JR, Marsh SA, Stevens JC, Rettie AE, McCarver DG, et al. Developmental Expression of Human Hepatic CYP2C9 and CYP2C19. The Journal of Pharmacology and Experimental Therapeutics. 2004 Mar;308(3):965–74. doi:10.1124/jpet.103.060137

16. Stajnko A, Runkel AA, Kosjek T, Snoj Tratnik J, Mazej D, Falnoga I, et al. Assessment of susceptibility to phthalate and DINCH exposure through CYP and UGT single nucleotide polymorphisms. Environment International. 2022 Jan;159:107046. doi:10.1016/j.envint.2021.107046

17. Bauer K, Versmold H, Prölss A, De Graaf SSN, Meeuwsen-Van Der Roest WP, Zijlstra WG. Estimation of Extracellular Volume in Preterm Infants <1500 g, Children, and Adults by Sucrose Dilution. Pediatr Res. 1990 Mar;27(3):256–9. doi:10.1203/00006450-199003000-00010

18. Vieux R, Hascoet JM, Merdariu D, Fresson J, Guillemin F. Glomerular Filtration Rate Reference Values in Very Preterm Infants. Pediatrics. 2010 May 1;125(5):e1186–92. doi:10.1542/peds.2009-1426

19. Ahn Y. Formula and scale for body surface area estimation in high-risk infants. Coll Antropol. 2010 Dec;34(4):1273–80. PubMed PMID: 21874709.

20. Astley ME, Chesnaye NC, Hallan S, Gambaro G, Ortiz A, Carrero JJ, et al. Age- and sex-specific reference values of estimated glomerular filtration rate for European adults. Kidney International. 2025 Jun;107(6):1076–87. doi:10.1016/j.kint.2025.02.025

21. Lopriore E. The total volume of blood in an extremely preterm neonate is about the size of a double espresso. Acta Paediatrica. 2023 Dec;112(12):2458–9. doi:10.1111/apa.16994

22. Wolfsberger CH, Seidel BN, Fleck L, Herrmann M, Kramer D, Benesch M, et al. Complete blood count reference intervals for extremely preterm neonates. Eur J Pediatr. 2025 Oct 18;184(11):699. doi:10.1007/s00431-025-06544-4 PubMed PMID: 41108422; PubMed Central PMCID: PMC12535521.

23. Tobias A, Ballard BD, Mohiuddin SS. Physiology, Water Balance. In: StatPearls (Internet). Treasure Island (FL): StatPearls Publishing; 2025 (cited 2026 Feb 7). Available from: http://www.ncbi.nlm.nih.gov/books/NBK541059/ PubMed PMID: 31082103.

Chapter S2 - BPA model

Compared to diisononyl phthalate (DINP) and other phthalate derivatives, the health risks of Bisphenol A (BPA) exposure are relatively well studied. However, regarding detailed human pharmacokinetic investigations, only two oral (1,2) and no parenteral dosing studies were identified.

Overview of BPA metabolism and enzymes involved

For modeling, we selected an oral dosing study by Thayer et al. as the most relevant (2). It showed that after oral administration of 100 µg/kg d6-BPA, over 99% of the applied dose is present as Phase II conjugates in serum with only negligible amounts of free compound detectable. The ratio of sulfated (BPA-S) to glucuronidated (BPA-G) BPA conjugates was determined to be 0.395 ($AUC_{(serum,\ BPA\text{-}S)}/AUC_{(serum,\ BPA\text{-}G)}$, excluding bis-conjugates) (2). While plasma half-lives ($t_{1/2(plasma)}$) of the conjugates might have been used for subsequent modeling, oral uptake and associated first-pass metabolism may differ from parenteral administration. A related study by the same group using transdermal d6-BPA administration suggests that as much as 8.8% of free BPA may be present in serum after parenteral exposure (3). Thus, first-pass metabolism in the intestine seems to contribute to the rapid clearance of free BPA from plasma after oral administration. Notably, only a minor fraction (< 5%) of free BPA is directly cleared by the kidneys, indicating that the observed "pool of free BPA" represents a consequence of an "overwhelmed" hepatic Phase II metabolism unsupported by intestinal metabolization (4). As for the molecular basis of BPA Phase II metabolism, glucuronidation predominantly occurs via UDP-glucuronosyltransferase 2B15 (UGT2B15) (5), whereas evidence points towards involvement of Sulfotransferase Family 1A Member 1 (SULT1A1) in sulfation (6).

Calculation of input concentrations for free- and conjugated BPA

Although data on dermal exposure to BPA suggest 8.8% of BPA to be present in serum in its free form (3), this value most likely underestimates the rapid rise of free BPA during intravenous administration due to slow-release kinetics from dermal compartments. Thus, we rely on a study of Kurebayashi et al. (7) and two studies by Doerge et al. (8,9), who investigated the pharmacokinetics of intravenous administration of 100 µg/kg BPA in cynomolgus monkeys and pregnant- and non-pregnant rhesus macaques. These studies identified 28% (cynomolgus monkeys), 29% (non-pregnant rhesus macaques), and 13% (pregnant rhesus macaques) of BPA present in its free form 5 min after intravenous application (7–9). Using only data on non-pregnant animals, we adopted a mean free-BPA fraction of 0.28 as a conservative modeling starting point. We did not scale this value to reflect the neonatal setting, as neonatal rhesus macaques showed similar kinetics (26% aglycone BPA after 5min) (8). Next, we separated the conjugated BPA into a sulfated and a glucuronidated fraction. In humans, this ratio can only be derived from oral administration data, thus, includes first-pass metabolism (2). To model conjugated compound fractionation in the parenteral setting, we

therefore had to use data on metabolite distribution in the urine (0-24h) measured from intravenously dosed cynomolgus monkeys (7). Based on these data we calculated a sulfated BPA fraction of 0.158. Thus, to reduce model complexity while accounting for the almost instantaneous conjugation of intravenously applied BPA, we modeled 72% of the administered BPA dose to be present in its conjugated form at time zero. Moreover, we partitioned the applied dosage of conjugated substance into 15.8% sulfated and 84.2% glucuronidated BPA. Consequently, we calculated influx as:

(1) $R_{in(free\ BPA)} = R_{in} * 0.28$

(2) $R_{in(BPA\text{-}G)} = (R_{in} * 0.72) * 0.842$

(3) $R_{in(BPA\text{-}S)} = (R_{in} * 0.72) * 0.158$

To calculate the bioactive, non-protein bound fraction of free BPA in infant blood, we used human experimental data on protein binding published by Gayrard et al. (10) and modeled the amount as $C_{(non\text{-}bound,\ free\ BPA)} = C_{(free\ BPA)} * 0.050$.

Metabolism and clearance of free BPA

Because the absorption $t_{1/2}$ calculated by Thayer et al. for aglycone BPA after oral administration to humans is rapid (0.52 h), we used the terminal $t_{1/2}$ measured in plasma of human adults ($t_{1/2}$ = 5.6 h) for the calculation of clearance of unconjugated BPA (2). We used this value instead of the terminal $t_{1/2}$ estimated from i.v. dosing of cynomolgus monkeys, as the value is likely more representative of human physiology. We adopted a $V_{d(free\ BPA)}$ value of 1.79 L/kg as proposed by Chapin et al. (11). Next, we calculated $CL_{(free\ BPA,\ adult)}$ and multiplied this value by the hepatic volume ratio (0.016) between adults (12) (1360 cm^3) and preterm infants (13) (21.13 cm^3) to calculate preterm clearance $CL_{(free\ BPA,\ preterm)}$. Due to the absence of additional experimental data on hepatic blood flow in prematurely born infants, we could not further account for hemodynamic differences that might influence this value. We then adapted $CL_{(free\ BPA,\ preterm)}$ using a metabolization coefficient of 0.158 and 0.842 for sulfated or glucuronidated BPA (7) and scaled respective clearances based on UGT2B15 and SULT1A1 protein expression ratios (UGT2B15 = 0.15, SULT1A1 = 0.42) measured from human hepatic tissue samples during maturation (14,15). Combining these considerations, we calculated the overall clearance of free BPA in preterm infants as $CL_{(free\ BPA,\ preterm,\ maturation\ scaled)} = (CL_{(free\ BPA,\ preterm)}*0.158)*MF_{(SULT1A1)} + (CL_{(free\ BPA,\ preterm)}*0.842)*MF_{(UGT2B15)}$.

Clearance of BPA Phase-II metabolites

The renal clearance of free BPA is negligible (4). In contrast, glucuronidated and sulfated BPA are cleared almost exclusively by the kidneys (7). Assuming terminal $t_{1/2}$ values of 5.5h and 4.4h estimated by Thayer et al. (2) after oral dosing of humans and V_{ss} values of 0.13 and 0.12 L/kg for BPA-G and BPA-S (16), respectively, we calculated the renal clearance of these compounds. Assuming neither tubular secretion nor reabsorption affects the elimination of these substances, we then scaled $CL_{(BPA\text{-}G,\ adult)}$ and $CL_{(BPA\text{-}S,\ adult)}$ to a 28-week-old preterm infant using the formula $CL_{(BPA\text{-}G/S,\ preterm)} = (GFR_{(28\text{-}weeks\ GA)}/GFR_{(adult)})*CL_{(BPA\text{-}G/S,\ adult)}$. For the GFR input of a model preterm infant, we used a value of 16.2 mL/min per 1.73 m^2, as measured by Vieux et al. seven days after birth in infants born at 28 weeks of gestation (17). To match the Thayer et al. cohort from which we derive $t_{1/2}$ values for respective Phase II metabolites, we used the GFR reference value (101.5 mL/min per 1.73 m^2) for healthy individuals aged 30-35 (mean age Thayer et al. = 32 years), as published by Astley et al. (18).

Compilation of reduced models

To understand possible differences in BPA pharmacokinetics related to prematurity, two reduced models were calculated. The first comprises all aspects of the "Preterm: full model" but omits additional scaling of $CL_{(free\ BPA)}$ by differences in UGT2B15 and SULT1A1 expression. This enables visualization of enzyme maturation effects on the elimination of $BPA_{(free)}$. The second model compares BPA pharmacokinetics between preterm infants and a 70-kg reference adult. In this model, the infusion rate was adjusted using the formula $Vin_{(adult,\ i)} = ((1/BW_{(day,\ i)}) * Vin_{(preterm,\ i)}) * 70$, to emulate equal daily exposure. Additionally, factors used to scale hepatic clearance of substances (e.g. liver volume and protein expression) and renal clearance were set to 1. Because adult clearances for a reference 70-kg human were used to inform our preterm infant calculations, omitting scaling factors and adjusting exposure rates creates an adult reference model suitable for comparison.

List of pharmacokinetic abbreviations and their respective units

CL	Clearance	L/h
V_d	Volume of distribution	L/kg or L, depending on calculation
V_{ss}	V_d at steady state	L/kg or L, depending on calculation
$t_{1/2}$	Half-life	hours
AUC	Area under the curve	n.a.
Rin	Xenobiotic influx rate	nmol/h
Vin	Infusion rate	mL/h

BW	Body weight	kg

References

1. Völkel W, Colnot T, Csanády GA, Filser JG, Dekant W. Metabolism and Kinetics of Bisphenol A in Humans at Low Doses Following Oral Administration. Chem Res Toxicol. 2002 Oct 1;15(10):1281–7.

2. Thayer KA, Doerge DR, Hunt D, Schurman SH, Twaddle NC, Churchwell MI, et al. Pharmacokinetics of bisphenol A in humans following a single oral administration. Environment International. 2015 Oct;83:107–15.

3. Sasso AF, Pirow R, Andra SS, Church R, Nachman RM, Linke S, et al. Pharmacokinetics of bisphenol A in humans following dermal administration. Environment International. 2020 Nov;144:106031.

4. Liu J, Martin JW. Prolonged Exposure to Bisphenol A from Single Dermal Contact Events. Environ Sci Technol. 2017 Sep 5;51(17):9940–9.

5. Hanioka N, Naito T, Narimatsu S. Human UDP-glucuronosyltransferase isoforms involved in bisphenol A glucuronidation. Chemosphere. 2008 Dec;74(1):33–6.

6. Nishiyama T, Ogura K, Nakano H, Kaku T, Takahashi E, Ohkubo Y, et al. Sulfation of Environmental Estrogens by Cytosolic Human Sulfotransferases. Drug Metabolism and Pharmacokinetics. 2002;17(3):221–8.

7. Kurebayashi H. Disposition of a Low Dose of Bisphenol A in Male and Female Cynomolgus Monkeys. Toxicological Sciences. 2002 Jul 1;68(1):32–42.

8. Doerge DR, Twaddle NC, Woodling KA, Fisher JW. Pharmacokinetics of bisphenol A in neonatal and adult rhesus monkeys. Toxicology and Applied Pharmacology. 2010 Oct 1;248(1):1–11.

9. Patterson TA, Twaddle NC, Roegge CS, Callicott RJ, Fisher JW, Doerge DR. Concurrent determination of bisphenol A pharmacokinetics in maternal and fetal rhesus monkeys. Toxicology and Applied Pharmacology. 2013 Feb;267(1):41–8.

10. Gayrard V, Viguie C, Cabaton N, Person E, Zalko D, Grandin F, et al. Importance of relative binding of bisphenol A and bisphenol S to plasma proteins for predicting their in vivo potencies. Toxicology and Applied Pharmacology. 2023 May;466:116477.

11. Chapin RE, Adams J, Boekelheide K, Gray LE, Hayward SW, Lees PSJ, et al. NTP-CERHR expert panel report on the reproductive and developmental toxicity of bisphenol A. Birth Defects Research Pt B. 2008 Jun;83(3):157–395.

12. Kromrey ML, Ittermann T, vWahsen C, Plodeck V, Seppelt D, Hoffmann RT, et al. Reference values of liver volume in Caucasian population and factors influencing liver size. European Journal of Radiology. 2018 Sep;106:32–7.

13. Szpinda M, Paruszewska-Achtel M, Woźniak A, Mila-Kierzenkowska C, Elminowska-Wenda G, Dombek M, et al. Volumetric Growth of the Liver in the Human Fetus: An Anatomical, Hydrostatic, and Statistical Study. BioMed Research International. 2015;2015:1–8.

14. Divakaran K, Hines RN, McCarver DG. Human Hepatic UGT2B15 Developmental Expression. Toxicological Sciences. 2014 Sep 1;141(1):292–9.

15. Dubaisi S, Caruso JA, Gaedigk R, Vyhlidal CA, Smith PC, Hines RN, et al. Developmental Expression of the Cytosolic Sulfotransferases in Human Liver. Drug Metabolism and Disposition. 2019 Jun;47(6):592–600.

16. Wiśniowska B, Linke S, Polak S, Bielecka Z, Luch A, Pirow R. Data on ADME parameters of bisphenol A and its metabolites for use in physiologically based pharmacokinetic modelling. Data in Brief. 2023 Jun;48:109101.

17. Vieux R, Hascoet JM, Merdariu D, Fresson J, Guillemin F. Glomerular Filtration Rate Reference Values in Very Preterm Infants. Pediatrics. 2010 May 1;125(5):e1186–92.

18. Astley ME, Chesnaye NC, Hallan S, Gambaro G, Ortiz A, Carrero JJ, et al. Age- and sex-specific reference values of estimated glomerular filtration rate for European adults. Kidney International. 2025 Jun;107(6):1076–87.

Chapter S3 - Additional Information on Data in Table 4

Search Strategy for Data Validation

A list of abbreviations can be found at the end of Chapter S3. The quality of documentation regarding HBGVs such as TDI, NOAEL, and DNEL for substances related to human health risk published by regulatory entities varies when compared to academic literature. Table 4 presents provisional parenteral HBGVs for adults and premature infants derived using assessment factors anchored in European guidelines and toxicological points of departure identified by international regulatory bodies.

The initial step involved document localization through a two-pronged approach: (i) collecting sources via search strategies based on human expertise, and (ii) independently using AI agents trained for biomedical research tasks. In cases of divergent information, documents were analyzed in depth until the value underlying the limit in effect was successfully verified, prioritizing regulatory body decisions over scientific literature.

Priority was given to documents from the EFSA, the ECHA, the EMA or, if unavailable, other officially recognized national/international regulatory bodies. Numerical values presented in the respective documents sometimes show slight divergence, despite intended harmonization. The databases and webpages of European regulatory bodies are similarly dynamic: documents may be removed over time without replacement or substituted with links to chemical safety data sheets. Consequently, a limited number of references had to cite publications from chemical producers and/or suppliers.

To allow a standardized approach to HBGV derivation, the EMA framework for PDE calculation was used, as proposed by the ICH Q3 guidelines. Specifically, the endpoints provided by the EFSA, the ECHA, or the EMA were researched for each compound of interest (where applicable) and toxicological point-of-departure (PoD) values for calculations identified from source documents. Subsequently, NOAEL or LOAEL were extracted from the cited primary literature and the route of administration was verified. These values were used to calculate permitted daily exposure (PDE) levels, according to the ICH-Q3C(R9) (1) guideline implemented by the EMA. As proposed in Appendix 3 of this document, five assessment factors (F1 to F5) were used in our calculations. Where applicable, oral to parenteral conversion assessment factors (F6) were implemented in line with the 2025 ICH-Q3E draft framework on extractables and leachables (2). As for prematurity-centered risk adaptation, no specific EMA guideline was identified. However, the EFSA proposed to introduce an additional safety factor of 3 for orally exposed term infants for substances with unknown pharmacokinetic profile or to use specific pharmacokinetic assessment factors for substances with known metabolic fate (3).

Since performing a detailed assessment for each substance is outside the scope of this paper, we adopted a pragmatic approach for the calculation of neonatal/preterm risk assessment factors. For

the two main contaminants observed in parenteral nutrition (DINP and BPA), for which pharmacokinetic models were calculated in Chapters S1 and S2, respectively, we applied an EFSA-informed "case-by-case" derivation: the ratio of AUC-values of bioactive molecules (parent compounds and non-conjugated metabolites) was calculated between preterm infants and adults and the calculated value used to inform the HBGV. HBGV values for all other substances were corrected using a sensitivity factor of 10 on top of the intraspecies factor (F2), as proposed by the US FQPA (FFDCA §408(b)(2)(C)) for pre- and postnatal toxicity of pesticides. This factor was deliberately selected as a conservative screening approach because the pharmacokinetic model predicted a 4.8-fold prematurity-specific toxicokinetic adjustment for BPA relative to adults, thus, exceeding the uncertainty factor of 3 proposed by the EFSA for substances with unclear metabolization (3). Accordingly, the 10-fold factor was chosen to provide an additional margin of conservatism for compounds for which prematurity-specific pharmacokinetic adjustments could not be derived.

Comments on Substances of Interest

Butyl benzyl phthalate (BBP) (4-7): the acceptable exposure value was reaffirmed in a 2019 update by the EFSA Panel on Food Contact Materials, Enzymes and Processing Aids (CEP Panel) (4). BBP was identified in the REACH Authorization List (Annex XIV) as a "Substance of Very High Concern" due to its endocrine-disruptive, reproductive, and developmental toxicity (5). The TDI of 0.50 mg/kg bw/d was implemented by the EFSA in 2019 due to the reprotoxic effects of the compound using a conversion factor of 100 (F1 = 10, F2 = 10). In line with ICH-Q3C(R9), we recalculated a PDE of 0.019 mg/kg bw/d (F1 = 5, F2 = 10, F3 = 1, F4 = 5, F5 = 1) for the index compound DEHP, using the EFSA-reviewed NOAEL of 4.8 mg/kg bw/d as a toxicological point of departure. Given the absence of maternal toxicity that explains observed testes-related toxicity at 300 ppm (NOAEL) in male F1 and F2 pups, a value of 5 was chosen for assessment factor F4 (6). Applying the EFSA approach, the derived value was divided by a relative potency factor of 0.1 to yield a final PDE of 0.19 mg/kg bw/d. In line with the 2025 ICH-Q3E draft framework on extractables and leachables, assuming an oral bioavailability of 100%, as adopted in a 2015 risk assessment performed by the Australian Industrial Chemicals Introduction Scheme (AICIS, formerly NICNAS) (7), no further parenteral correction factor was applied (F6 = 1). The intermediate result of 0.192 mg/kg bw/d was further divided by 10, an FQPA-informed sensitivity factor, yielding a final, provisional HBGV of 0.0192 mg/kg bw/d.

Diethyl phthalate (DEP) (8-11): under EU regulations, DEP is currently not classified as an endocrine disruptor or reproductive toxicant. DEP has a lower acute toxicity than BBP. To the best of our knowledge, no TDI or DNEL for DEP has been proposed by the EFSA or the ECHA. However, an EMA document published in 2014 identified a PDE of 4.0 mg/kg bw/d for DEP. This value was calculated based on a two-generation reproductive toxicity study in rats that reported a NOAEL of 197 mg/kg bw/d for effects on the development and growth of pups by applying a conversion factor

of 50 (F1 = 5, F2 = 10). In line with the ICH-Q3C(R9) guideline and using the same NOAEL as a PoD, we calculated a PDE of 0.79 mg/kg bw/d (F1 = 5, F2 = 10, F3 = 1, F4 = 5, F5 = 1). The value of 5 for F4 was chosen since effects on liver, adrenal gland, and epididymis in the F0 generation at 1016 mg/kg bw/d DEP either specifically pertained to male animals or were considered toxicologically irrelevant by the authors (10). Assuming 100% oral bioavailability, as adopted in a 2011 risk assessment by the AICIS (11), no parenteral correction factor was applied (F6 = 1). The PDE was further divided by an FQPA-informed sensitivity factor of 10, yielding our final provisional HBGV of 0.079 mg/kg bw/d.

Diisononyl phthalate (DINP) (4, 12, 13): Although DINP is considered less toxic than DEHP, it remains strictly regulated in childcare appliances due to potential developmental and liver toxicity. To our knowledge, no EMA-proposed HBGV exists. However, a 2019 EFSA risk assessment update regarding phthalates in food contact materials reaffirmed the relevance of DINP-induced liver toxicity over reproductive effects. The document states "The Panel noted that DINP and DIDP affect the liver at doses lower than those associated with their reproductive effects, as indicated by the NOAELs for reproductive effects reported below. For this reason, the individual TDIs for DINP and DIDP of 0.15 mg/kg bw per day remain based on the liver effects." (4). Consequently, we used the EFSA-derived NOAEL from the unpublished Exxon study and a study by Lington et al. (13). Both investigations analyzed the effects of oral DINP administration to Fischer 344 rats over a two-year exposure duration. Hepatic and renal weight increases were documented in both studies at NOAELs of 15 mg/kg bw/d (Exxon study) and 17 mg/kg bw/d (Lington et al.), respectively. Thus, 15 mg/kg bw/d served as the PoD for our calculations. Due to the absence of human bioavailability data for DINP, absorption data from rat studies presented in Chapter S1 (mean absorption about 82%) were employed to calculate the parenteral conversion factor (F6 = 2). After conversion of the NOAEL to 7.5 mg/kg bw/d, an HBGV of 0.063 mg/kg bw/d (F1 = 5, F2 = 24, F3 = 1, F4 = 1, F5 = 1) was calculated for DINP. The default toxicokinetic dispersion factor F2 (3.16) was retained to account for within-population variability and multiplied by a compound-specific factor (2.4) for preterm infant exposure. This factor represents the AUC ratio of bioactive compounds between preterm- and adult pharmacokinetic models. The toxicodynamic sub-component was retained at its default value (3.16). This methodology, adopted from Appendix C of the 2017 EFSA guideline on the risk assessment of food contaminants in children below 16 weeks of age (3), yielded a final F2 factor of 24. Factors of 1 were assigned for F3 and F4, respectively, as the exposure duration comprised at least half the species' lifespan and no severe toxicity was observed.

Dimethyl phthalate (DMP) (14-17): in rodents (mouse, rat, guinea pig), DMP has low chronic toxicity compared to long-chain phthalates like DINP or butyl phthalate. Acute toxicity (LD50) is thought to occur only at extreme doses (2900-10100 mg/kg). DMP is currently not regulated by REACH and is listed by the EPA as a Group D chemical (i.e., not classifiable for carcinogenicity). A Hazard Assessment Report published by the Australian Government identified a NOAEL of 840 mg/kg bw/d

for maternal toxicity (weight loss, reduced weight gain during treatment) from a study that exposed pregnant Sprague-Dawley rats orally to DMP between gestational day 6 and 15. Using this NOAEL as a PoD, we calculated a PDE of 17 mg/kg bw/d using an EMA-based approach (F1 = 5, F2 = 10, F3 = 1, F4 = 1, F5 = 1). Assessment factors F3 and F4 were set to 1 since (i) organogenesis in Sprague-Dawley rats is usually finished by GD 16 and dosing was performed between GD6 and 15, (ii) embryos were analyzed at GD 20, and (iii) no carcinogenic, neurological or teratogenic effects were observed in the study (15). Next, an oral bioavailability of 100% for DMP was adopted, in line with a 2014 Chemical Assessment Report issued by the AICIS (16) and a summary report on DMP and DEP by the European Agency for the Evaluation of Medicinal Products (EMEA) (17). Considering the 2025 ICH-Q3E draft framework on extractables and leachables, no further parenteral correction factor was applied (F6 = 1). Like before, a pragmatic FQPA-informed sensitivity factor of 10 was applied to the value, yielding a final provisional HBGV of 1.7 mg/kg bw/d.

Monomethyl phthalate (MMP): no regulatory body documents specifically addressing toxicity or other adverse effects of MMP could be identified. Additionally, data on the parent compound DMP suggest limited toxicity. Thus, the substance was omitted from further investigation.

Monobutyl-/Monoisobutyl phthalate (MnBP/MiBP) (4, 18-21): no specific DNEL or TDI levels are available since the substances are in vivo metabolites of dibutyl phthalate/diisobutyl phthalate (DBP/DiBP). The parent substances, however, are significant reproductive (male) and developmental toxicants as well as endocrine disruptors. They are listed as “substances of very high concern” by the EU and are banned from childcare articles. DNEL reference values have been proposed by the ECHA for the parent compounds DBP (0.0067 mg/kg bw/d) and DiBP (0.0083 mg/kg bw/d). Importantly, the ECHA used an uncertainty factor of 10 for interspecies differences (rats), a factor of 10 for intraspecies differences, and a factor of 3 for conversion of LOAEL values to derive these values. Assessment factors for exposure duration were not considered. Underlying LOAEL values were derived from a developmental study that showed effects of DBP on mammary gland development in offspring after oral dosing of adult female rats from GD 15 to PND 21. Values for DiBP were calculated from DBP-specific thresholds using a read-across approach. In contrast, our calculation based on the same LOAELs for DBP (= 2 mg/kg bw/d) and DiBP (= 2.5 mg/kg bw/d) yielded a provisional HBGV of 0.00027 mg/kg bw/d for DBP and 0.00033 mg/kg bw/d for DiBP (F1 = 5, F2 = 10, F3 = 10, F4 = 5, F5 = 3). An assessment factor of 10 was chosen for F3 in line with the ICH-Q3C(R9) guideline because the study from which the toxicological PoD was derived dosed rats after the critical window of organ maturation and had a maximal exposure duration of less than three months. A value of 5 was used for F4 despite the presence of significantly reduced weight gain in maternal animals between GD 15 and GD 20 at 20 ppm exposure (LOAEL). Our reasoning for this choice was that toxicological findings in maternal animals were not dose-dependent and developmental effects on offspring were mechanistically distinct (20). Further, an F5 value of 3 was chosen due to the absence of a NOAEL value, in line with the penalization performed by the ECHA.

Next, considering the 2025 ICH-Q3E draft framework on extractables and leachables and an oral dosing study with deuterated DiBP and DnBP in a single human male volunteer (urinary excretion of DiBP/DnBP and related metabolites > 90% / 48h), no parenteral modifier was applied to the provisional HBGV of DiBP (F6 = 1). Based on the same publication, no parenteral correction factor was applied to the provisional HBGV of DBP either (F6 = 1) (21). Values were subsequently adjusted to equivalent mono-ester mass doses (conversion factor = 0.80), yielding a provisional HBGV of 0.00022 mg/kg bw/d for MnBP and 0.00026 mg/kg bw/d for MiBP. This calculation assumes complete conversion of the parent compounds to the monoesters, thus, is inherently limited by this assumption. A pragmatic FQPA-informed sensitivity factor of 10 was applied to the value. This results in a final provisional HBGV of 0.000022 mg/kg bw/d for MnBP and 0.000026 mg/kg bw/d for MiBP.

N-butylbenzenesulphonamide (NBBS) (22, 23): NBBS exhibits developmental, reproductive, and potential neurotoxic effects, along with liver/kidney impairment in rodents. It is a known groundwater contaminant. For calculation of a provisional HBGV, toxicological data in a 2010 REACH Dossier from a repeated-dose toxicity study were used. This study investigated oral treatment of Wistar rats with NBBS over a 90-day exposure. A NOAEL for effects on body weight, kidney enlargement, and pathological abnormalities in liver tissue was reported at 51.9 mg/kg bw/d. This value was used as the PoD for our calculations. Due to the absence of oral bioavailability data in humans, the study by Waidyanatha et al. (22) was used as a surrogate starting point. The authors investigated oral absorption of NBBS in HSD rats at comparable doses to the study cited in the REACH Dossier (NOAEL = 51.9 mg/kg vs. 60 mg/kg), observing a mean absorption of 60% (calculated as (68+52)/2). In line with the 2025 ICH-Q3E draft framework on extractables and leachables, the respective oral NOAEL was modified by a parenteral correction factor (F6 = 2) to 26 mg/kg bw/d. As above, the ICH-Q3C(R9) guideline-conform safety stack calculations were subsequently applied to this value. Thus, a revised provisional HBGV of 0.10 mg/kg bw/d was calculated for NBBS (F1 = 5, F2 = 10, F3 = 5, F4 = 1, F5 = 1). Values of 5 and 1 were introduced for assessment factors F3 and F4 to account for study length (90 days) and absence of severe toxicity (e.g., neurotoxicity, carcinogenicity, teratogenicity) (23). The calculated intermediate result was further divided by an FQPA-informed sensitivity factor of 10 to yield a provisional HBGV of 0.010 mg/kg bw/d.

Bisphenol A (BPA) (3, 24-31): BPA is an endocrine disruptor present in plastics and food can liners. It is associated with reproductive dysfunction, immune dysregulation, metabolic disorders (e.g., diabetes, obesity), cognitive impairment, and increased cancer risk. In 2023, the EFSA issued a report in which the previous TDI (proposed in 2015) of 4 µg/kg bw/d was lowered 20,000-fold to 0.2 ng/kg bw/d. This adjustment was based on immunotoxic effects, specifically increased abundance of TH17 cells in the spleens of mice exposed to BPA during gestation. This proposed value was not endorsed by the EMA and diverged significantly from the value derived by the German Federal Institute for Risk Assessment (BfR). The discrepancy prompted the publication of two joint

documents clarifying the scientific divergence (27, 28). For the EMA to accept intermediary endpoints (e.g., elevated TH17 cell percentages) as a PoD for risk assessment, a causal link to an apical endpoint, identified within the design and scope of a study must be established. Conversely, the EFSA considers causal links inferred through weight-of-evidence assessments. Consequently, the EMA considers the evidence used by the EFSA for BPA HBGV calculation insufficient to establish a direct causal and harmful effect in humans. Differences in PoD selection criteria likely reflect the distinct mandates of the respective regulatory bodies. While the EMA quantifies toxicity risks associated with defined substance application, the EFSA is tasked to investigate risks by lifetime dietary exposure to the general population. Thus, the EFSA approach is more restrictive than that of the EMA. A joint statement issued by the BfR and the EFSA also noted discrepancies in PoD selection and questioned the EFSA's quality grading of included studies and the proposed human equivalent dose factor (HEDFs) calculations. Given that (i) our methodological framework for HBGV calculation is anchored in EMA guidelines, (ii) the EMA does not provide a defined HBGV for BPA, and (iii) the BfR-derived HBGV aligns more closely with EMA guidelines than the EFSA-calculated value, we adopted the PoDs proposed by the BfR for HBGV calculation (29). Specifically, the BfR identified two studies that investigated the effects of oral BPA treatment on sperm counts in adult Wistar rats as pertinent to PoD determination (30, 31). Thus, using a NOAEL of 20 µg/kg bw/d, as proposed by Liu et al., and a NOAEL of 50 µg/kg bw/d, as proposed by Srivastava et al., we calculated provisional HBGVs of 8.33 ng/kg bw/d (F1 = 5, F2 = 48, F3 = 10, F4 = 1, F5 = 1) and 21 ng/kg bw/d (F1 = 5, F2 = 48, F3 = 10, F4 = 1, F5 = 1) for oral BPA exposure. The default toxicokinetic dispersion factor F2 (3.16) was retained to account for within-population variability and multiplied by a compound-specific factor (4.8) for preterm infant exposure. This factor was derived as the AUC ratio of bioactive compounds between preterm and adult population-specific pharmacokinetic models. The toxicodynamic sub-component remained at its default value (3.16). This procedure, adopted from Appendix C of a 2017 EFSA guideline on the risk assessment of food contaminants in children below 16 weeks of age (3), yielded a final F2 factor of 48. A value of 10 was assigned for F3, as the exposure duration of both studies was less than three months. A value of 1 was assigned for F4, as both studies investigated effects on male rats outside the critical window of development. Due to the high absorption of BPA, no parenteral correction factor (F6 = 1) was applied. These calculations yielded final HBGVs of 8.33 ng/kg bw/d and 21 ng/kg bw/d for the two PoDs proposed by the BfR.

<u>Bisphenol S (BPS)</u> (32-35): High exposure to BPS is possible from handling thermal printer paper. In animal studies, BPS intake was shown to exhibit similar toxicity as BPA. In 2020, the EFSA was tasked by the European Commission to reassess the specific migration limit (SML) of BPS based on new toxicological studies. It initially concluded that no change in the SML of 0.05 mg/kg food was necessary. This was recently superseded by the EU, putting BPS under the same ban as BPA (Regulation (EU) 2024/3190, effective 20 January 2025). However, we were unable to derive a

distinct HBGV for BPS. Thus, we used the lowest NOAEL included in the 2020 EFSA technical report on BPS toxicity for the calculation of a provisional PDE. The NOAEL (20 mg/kg bw/d) value pertained to developmental immunotoxicity and post-implantation loss observed in rats upon oral BPS exposure. We adapted the NOAEL-value using an ICH-Q3C(R9)-style approach, arriving at a provisional HBGV of 0.4 mg/kg bw/d for BPS (F1 = 5, F2 = 10, F3 = 1, F4 = 1, F5 = 1). F4 was set to 1 due to the congruent NOAEL for post-implantation loss (maternal effect) and developmental immunotoxicity (offspring effect). A pharmacokinetic investigation by Oh et al. in which 8.75 µg/kg bw of deuterated BPS were orally administered to 7 healthy individuals reported a mean fractional excretion of 82% within 48h (35). Thus, we further adjusted our calculated value by division with a parenteral conversion factor (F6 = 2) to 0.2 mg/kg bw/d, according to the 2025 ICH-Q3E draft framework. This value was then finally corrected to a provisional HBGV of 0.02 mg/kg bw/d by pragmatically applying an FQPA-informed sensitivity factor of 10.

<u>Methylparaben (MP)</u> (36-42): According to the EFSA, MP has a provisional safety level allowing intake of up to 10 mg/kg bw/d (ADI) as the sum of methyl- and ethylparaben. Propylparaben was explicitly excluded from this threshold due to effects on sex hormones and reproductive organs of juvenile rats. To the best of our knowledge, commonly cited sources for the EFSA ADI calculation are a study by Matthews et al. (40), who reported a NOAEL of 1000 mg/kg bw/d, based on 96 week-long oral exposure of rats to the chemical (36) as well as an investigation by Hoberman et al., who found similar NOAEL values for reproductive toxicity in rats after oral treatment for up to 57 days (39). Additionally, a study by Oishi et al., published in 2004, also reported a NOAEL for reprotoxic effects of MP of 1000 mg/kg bw/d after oral exposure of rats for 8 weeks (41). Interestingly, an official report on MP was published by the Scientific Committee on Consumer Safety (SCCS). It evaluated the safety of MP as a cosmetic preservative, concluding that it is safe at a concentration of up to 0.4%. To derive this threshold, toxicological data from orally exposed animals were reviewed. The SCCS used an oral NOAEL of 300 mg/kg bw/d as the toxicological point of departure for their MoS calculations as a 2021 EOGRTS suggested anti-androgenic effects of methylparaben in F2 pups (reduced anogenital distance). Thus, using the same NOAEL as PoD for subsequent calculations we derived a provisional PDE of 1.2 mg/kg bw/d for MP (F1 = 5, F2 = 10, F3 = 1 F4 = 5, F5 = 1). An assessment factor of 5 was chosen for F4 due to the presence of anti-androgenic effects in offspring without sufficient maternal pathology. Since an oral dosing study with three healthy volunteers using deuterated MP reported a mean 48-hour urinary recovery of 84% as MP and related metabolites (42), our provisional HBGV was additionally divided by a parenteral conversion factor (F6 = 2). This resulted in a parenteral value of 0.6 mg/kg bw/d when applying the 2025 ICH-Q3E draft framework. As a pragmatic choice, this provisional HBGV was further divided by 10, an FQPA-informed sensitivity factor, yielding a final provisional HBGV of 0.06 mg/kg bw/d.

<u>Propylparaben (PP)</u> (36, 37, 43-48): In 2015 the EMA proposed a PDE of 2 mg/kg bw/d for adult and pediatric patients based on data that indicated estrogenic activity of propylparaben in rats after oral

force feeding. This value was calculated from a NOAEL of 100 mg/kg bw/d, as proposed by an unpublished report by Pouliot et al. (46) often cited in governmental reports, whose results apparently have been shared in Sivaraman et al. (47). As the EMA-proposed PDE uses the same underlying logic as our calculated metrics (F1 = 5, F2 = 10, F3-to-5 = 1), we use it for subsequent modeling of parenteral exposure in infants. A pharmacokinetic investigation by Shin et al. in which deuterated PP was orally administered to 12 healthy individuals, calculated an absorption of 39% from fractional urinary excretion (48). Thus, in line with the 2025 ICH-Q3E draft, we further adjusted the PDE of 2 mg/kg bw/d by division with a parenteral conversion factor (F6 = 10) to 0.2 mg/kg bw/d. This provisional HBGV was further adapted to 0.02 mg/kg bw/d using an FQPA-informed sensitivity factor of 10.

Perfluorobutane sulfonic acid (PFBS) (49-56): Although a TWI reference value of 4.4 ng/kg bw/week has been issued by the EFSA for the sum of 4 PFAS compounds PFOA, PFNA, PFHxS and PFOS, no defined threshold exists for PFBS as a single compound. However, a report on drinking water PFBS thresholds published by the EPA reports chronic reference doses (RfD) for PFBS of 0.3 µg/kg bw/d as its starting point for the calculation of lifetime noncancer drinking water Health Advisory (HA) values. This RfD stems from a PFBS toxicity assessment published by EPA in 2021 (54). This assessment uses perturbation of thyroid function, as observed in a study conducted by Feng et al., as their respective PoD (55). Feng et al. observed disruption of thyroid hormone activity, delays in development and pubertal onset, as well as disruption of endocrine-hormonal circuits in offspring mice after exposure of the mother to a minimum of 200 mg/kg bw/d PFBS throughout gestational days 1 to 20. Thus, a NOAEL of 50 mg/kg bw/d may serve as PoD for our calculations. Following the ICH-Q3C(R9) guidelines, we recalculated the PDE for PFBS as 0.083 mg/kg bw/d (F1 = 12, F2 = 10, F3 = 1, F4 = 5, F5 = 1). A value of 1 was chosen for F3, as administration of PFBS during GD 1 to 20 adequately covers organogenesis in ICR mice. A conservative value of 5 was selected for F4, because it is unclear whether the perturbation of thyroid function in dams at 200 mg/kg bw/d PFBS sufficiently explains intergenerational effects observed in the offspring. From a recent study in which a single human volunteer ingested 15 different mass-labeled PFAS compounds, an absorption of 100% can be assumed for most of the compounds (56). Thus, in line with the 2025 ICH-Q3E draft framework no further parenteral correction factor (F6 = 1) was applied. However, an FQPA-informed sensitivity factor of 10 was used, yielding a provisional HBGV of 0.0083 mg/kg bw/d.

5-hydroxymethyl-2-furanoic acid (5-HMFA) (57, 58): As a secondary derivative of 5-HMF (5-hydroxymethylfurfural), 5-HMFA is produced during heat sterilization of glucose-containing solutions. It is considered non-toxic by regulatory bodies, although the parent compound 5-HMF is slightly toxic with a NOAEL of 80-100 mg/kg bw/d. In consequence, the substance was omitted from further investigations.

Daidzein and Genistein (59-63): Both substances are soybean-derived phytoestrogen/isoflavone components contained within the lipid emulsion used for parenteral nutrition. Although toxicological

endpoints of an array of studies have been summarized by the Scientific Committee on Consumer Safety (SCCS), official regulatory documents only address their use in cosmetic products. However, based on data summarized in the SCCS document, we could derive provisional HBGV values for Daidzein and Genistein. To this end we adopted the PoD (NOAEL-equivalent value = 5 mg/kg bw/d) proposed by the SCCS based on the absence of direct effects of Daidzein on the fertility of male rats after subcutaneous administration of 5mg/kg bw/d for 50 days. For Genistein we used the proposed NOAEL of 4 mg/kg bw/d for estrogenic effects on developing female rat pups after combined subcutaneous and oral dosing as PoD for our calculations. The SCCS considered the abovementioned PoDs to be sufficiently conservative to account for potential endocrine-related effects. Based on the available data, we calculated provisional HBGV values for Genistein (F1 = 5, F2 = 10, F3 = 10, F4 = 1, F5 = 1) and Daidzein (F1 = 5, F2 = 10, F3 = 10, F4 = 1, F5 = 1) as 0.008 mg/kg bw/d and 0.01 mg/kg bw/d, respectively, using the ICH-Q3C(R9) guideline. F3 was set to 10 due to total exposure times in the respective PoD-informing studies lying below 3 months (21 days for Genistein and 50 days for Daidzein) (62, 63). Since (i) no compound-specific data on subcutaneous bioavailability of Genistein or Daidzein could be identified, and (ii) the SCCS assumed complete bioavailability following subcutaneous administration, a parenteral conversion factor (F6) of 1 was applied. The factor accounts for total systemic availability but not for route-dependent differences in concentration profiles over time. This particularly refers to lower peak concentrations and delayed absorption associated with subcutaneous depot release compared to intravenous administration. Therefore, the resulting conversion only partially captures pharmacokinetic differences between the two routes. Further adjustment would require compound-specific pharmacokinetic data and was beyond the scope of our assessment. Finally, the values were further adapted using a pragmatic sensitivity factor of 10 based on FQPA guidelines, yielding a final provisional HBGV of 0.001 mg/kg bw/d for Daidzein and 0.0008 mg/kg bw/d for Genistein.

<u>Methyl protocatechuic acid</u> (MPCA) (64): MPCA shows a beneficial anti-inflammatory effect, with no regulatory restrictions imposed on its use. No toxicity was observed at 40 mg/kg bw/d. Thus, the substance was omitted from further investigation. However, protocatechuic acid on its own (sans methyl) is problematic.

Limitations

Since data on fractional urinary excretion (for which several of the proposed provisional HBGV values were corrected) often lack comparisons to intravenous administration and may be confounded by biliary secretion, tissue retention, and urinary kinetics, the absorption data used to inform F6 selection do not constitute absolute estimates of bioavailability. Thus, default F6 conversion factors, as proposed in the ICH-Q3E Guideline, had to be applied to the available data. These assessment factors do not account for route-dependent differences in substance distribution- and concentration-time profiles, including organ-specific peak concentrations. Moreover, the use of an FQPA-informed sensitivity factor of 10 constitutes a pragmatic and conservative choice for risk

assessment. It is likely more conservative than a substance-specific pharmacokinetic adjustment, given that the corresponding assessment factors derived for DINP and BPA were 2.4 and 4.8, respectively. However, the approach was chosen to provide an additional margin of safety for compounds for which prematurity-specific pharmacokinetic adjustment could not be derived. Importantly, this did not affect the overall risk assessment, as no substance other than BPA exceeded its calculated HBGV. Due to the possibility for double-counting of assessment factors, values included in Table 4 of the main text are presented in their original format (adult, oral) as well as their recalculated form (neonatal, parenteral). The HBGV values shown are guideline- and literature-based proxies for the particularly vulnerable population of preterm infants rather than an update of existing guideline values proposed by dedicated working- and study groups. While we reviewed NOAEL-endpoints on a case-to-case basis for each compound, we refrained from addressing potential novel outcome measures (e.g., immunometabolic disruption, neurotoxicity) to limit divergence from official guidelines. Together, the values in this chapter should not be interpreted as advocacy for updates or replacements of regulator-issued HBGVs but be viewed as an honest attempt to integrate existing knowledge into a comprehensive framework.

Abbreviations

ADI – Acceptable Daily Intake; used for substances added to products. Estimate of dose that can be ingested daily over a lifetime without significant health risks.

AICIS – Australian Industrial Chemicals Introduction Scheme, formerly NICNAS

AUC – Area under the Curve (pharmacokinetic exposure measure)

BfR – German Federal Institute for Risk Assessment; https://www.bfr.bund.de/en/

CEP – Panel on Food Contact Materials, Enzymes and Processing Aids (EFSA)

DNEL – Derived No-Effect Level; the level of exposure to a substance above which humans should not be exposed as defined by REACH, ECHA and other bodies; https://www.dguv.de/ifa/gestis/gestis-dnel-liste/index.jsp

ECHA – European Chemicals Agency; manages chemical substance registration, evaluation, and release; https://echa.europa.eu/about-us

EFSA – European Food Safety Authority; https://www.efsa.europa.eu/en

EMA – European Medicines Agency; https://www.ema.europa.eu/en/homepage

EMEA – European Agency for the Evaluation of Medicinal Products; now EMA

EOGRTS – Extended One-Generation Reproductive Toxicity Study

EPA – U.S. Environmental Protection Agency; https://www.epa.gov/

F1-F6 – Assessment Factors used for HBGV calculation

FFDCA – Federal Food, Drug, and Cosmetic Act (USA)

FQPA – Food Quality Protection Act (USA)

GD – Gestational Day

HBGV – Health Based Guidance Value

HEDF – Human Equivalent Dose Factor

ICH-Q3C – International Council for Harmonisation (guideline for Impurities); Residual Solvents.

LD50 – Lethal Dose, 50%; represents the dose of a substance that kills 50% of test subjects (usually mice/rat). Expressed in mg/kg.

LOAEL – Lowest Observed Adverse Effect Level; the lowest tested dose of a substance that shows a significant toxicological effect on a defined health outcome.

MoS – Margin of Safety

NICNAS – National Industrial Chemicals Notification and Assessment Scheme

NOAEL – No Observed Adverse Effect Level; highest tested dose at which no adverse effect was observed under the study conditions; for reference see e.g. Chen C, Lavezzi SM, McDougall D. The estimation and translation uncertainties in applying NOAEL to clinical dose escalation. Clin Transl Sci. 2024;17(6):e13831. https://www.ncbi.nlm.nih.gov/pubmed/38808564

PDE – Permitted Daily Exposure; dose of a substance that is unlikely to cause adverse health effects in individuals exposed to it on a daily basis.

PND – Postnatal day

PoD – Toxicological Point of Departure; starting point used for the calculation of exposure limits such as DNEL, or RfD.

REACH – Registration, Evaluation, Authorization, and Restriction of Chemicals; EU regulation/framework; https://environment.ec.europa.eu/topics/chemicals/reach-regulation_en

RfD – Reference Dose; daily oral exposure that is unlikely to elicit a non-cancer health effect over a lifetime.

SCCS – Scientific Committee on Consumer Safety (SCCS); https://health.ec.europa.eu/scientific-committees/scientific-committee-consumer-safety-sccs_en

SML – Specific Migration Limit; maximum quantity of a substance allowed to migrate from food packaging into food.

TDI – Tolerable Daily Intake; amount of substance that can be taken daily over a lifetime without appreciable health risk.

TWI – Tolerable Weekly Intake; amount of substance that can be taken weekly over a lifetime without appreciable health risk.

WHO – World Health Organization; https://www.who.int/

References

1. (ICH) ICfH. ICH Q3C (R9) Guideline on impurities: guideline for residual solvents. In: Agency EM, editor. Amsterdam: European Medicines Agency; 2024.
2. (ICH) ICfH. ICH Q3E Guideline for extractables and leachables. In: Agency EM, editor. Amsterdam2025.
3. Committee ES, Hardy A, Benford D, Halldorsson T, Jeger MJ, Knutsen HK, et al. Guidance on the risk assessment of substances present in food intended for infants below 16 weeks of age. Efsa Journal. 2017;15(5):e04849.
4. EFSA Panel on Food Contact Materials E, Aids P, Silano V, Barat Baviera JM, Bolognesi C, Chesson A, et al. Update of the risk assessment of di-butylphthalate (DBP), butyl-benzyl-phthalate (BBP), bis (2-ethylhexyl) phthalate (DEHP), di-isononylphthalate (DINP) and di-isodecylphthalate (DIDP) for use in food contact materials. EFSA journal. 2019;17(12):e05838.
5. (ECHA) ECA. Benzyl butyl phthalate (BBP) — Authorisation process [ECHA CHEM database entry]. European Chemicals Agency; 2026 [Available from: https://chem.echa.europa.eu/100.001.475/activities/authorisationProcess/740e2853d69f7faa1692030898f8d8ab.
6. Program NT. Diethylhexyl phthalate: multigenerational reproductive assessment by continuous breeding when administered to Sprague-Dawley rats in the diet. Research Triangle Park, NC, NTP Study Number: RACB98004. 2004.
7. (NICNAS) NICNaAS. Priority Existing Chemical Assessment Report No. 40: Butyl Benzyl Phthalate (BBP). Sydney: Australian Government Department of Health 2015. Report No.: PEC40 Contract No.: ISBN 978-0-9758470-0-8.
8. (SCCNFP) SCoCPaN-FP. Opinion of the Scientific Committee on Cosmetic Products and Non-Food Products Intended for Consumers Concerning Diethyl Phthalate. European Commission; 2002 4 June 2002. Report No.: SCCNFP/0411/01.
9. (CHMP) CfMPfHU. Guideline on the Use of Phthalates as Excipients in Human Medicinal Products. Corrigendum 2 (Section 6.3 correction; addition of Annex I). European Medicines Agency 20 November 2014. Report No.: EMA/CHMP/SWP/362974/2012 corr 2.
10. Fujii S, Yabe K, Furukawa M, Hirata M, Kiguchi M, Ikka T. A two-generation reproductive toxicity study of diethyl phthalate (DEP) in rats. The Journal of toxicological sciences. 2005;30(Special):S97-116.
11. (NICNAS) NICNaAS. Priority Existing Chemical Assessment Report No. 33: Diethyl Phthalate. Australian Government Department of Health November 2011. Report No.: PEC33.
12. United States Environmental Protection Agency (EPA) OoCSaPP. Risk Evaluation for Diisononyl Phthalate (DINP). Washington, DC January 2025. Report No.: EPA-740-R-25-001.
13. Lington AW, Bird MG, Plutnick RT, Stubblefield WA, Scala RA. Chronic toxicity and carcinogenic evaluation of diisononyl phthalate in rats. Fundamental and applied toxicology. 1997;36(1):79-89.
14. (NICNAS) NICNaAS. Existing Chemical Hazard Assessment Report: Dimethyl Phthalate. Sydney: Australian Government June 2008.
15. Field EA, Price CJ, Sleet RB, George JD, Marr MC, Myers CB, et al. Developmental toxicity evaluation of diethyl and dimethyl phthalate in rats. Teratology. 1993;48(1):33-44.

16. (NICNAS) NICNaAS. Priority Existing Chemical Assessment Report No. 37: Dimethyl Phthalate. Sydney: Australian Government January 2014. Report No.: PEC37.
17. (CVMP) CfVMP. Dimethyl and Diethyl Phthalates: Summary Report. London: European Agency for the Evaluation of Medicinal Products (EMEA) September 2001 (corrigendum). Report No.: EMEA/MRL/049/95-CORRIGENDUM.
18. Khurshid S, Anjum M, Nawaz R, Kauser F, Saleem S, Hanafiah MM, Abbas M. Di-butyl phthalates (DBP) in the environment: health risks and advances in treatment technologies. Environmental Geochemistry and Health. 2025;47(9):371.
19. (ECHA) ECA. Annex XV Restriction Report: Proposal for a Restriction — Four Phthalates (DEHP, BBP, DBP, DIBP). Helsinki: Danish Environmental Protection Agency (collaborating) 1 April 2016.
20. Lee K-Y, Shibutani M, Takagi H, Kato N, Takigami S, Uneyama C, Hirose M. Diverse developmental toxicity of di-n-butyl phthalate in both sexes of rat offspring after maternal exposure during the period from late gestation through lactation. Toxicology. 2004;203(1-3):221-38.
21. Koch H, Christensen K, Harth V, Lorber M, Brüning T. Di-n-butyl phthalate (DnBP) and diisobutyl phthalate (DiBP) metabolism in a human volunteer after single oral doses. Archives of toxicology. 2012;86(12):1829-39.
22. Waidyanatha S, Gibbs S, South N, Smith JP, Mutlu E, Burback B, et al. Toxicokinetics of the plasticizer, N-butylbenzenesulfonamide, in plasma and brain following oral exposure in rodents: Route, species, and sex comparison. Toxicology reports. 2020;7:711-22.
23. Agency EC. N-butylbenzenesulphonamide (EC 222-823-6, CAS 3622-84-2): registered substance dossier [Internet]. Helsinki: European Chemicals Agency; [Available from: https://chem.echa.europa.eu/100.020.749/dossier-view/7909b8f3-3fcd-42d7-85d4-592e165907d7/0d8800f2-caf6-499b-92f5-cd599de77ff6_49b20a68-05f1-4a44-8d1d-6f335303453a?searchText=N-butylbenzenesulphonamide.
24. Commission E. Commission Regulation (EU) 2024/3190 of 19 December 2024 on the use of bisphenol A (BPA) and other bisphenols and bisphenol derivatives with harmonised classification for specific hazardous properties in certain materials and articles intended to come into contact with food, amending Regulation (EU) No 10/2011 and repealing Regulation (EU) 2018/21. Off J Eur Union. 2024:2024/3190.
25. Carreño García I, Dolle T. The European Commission prohibits Bisphenol A and other bisphenols in food contact materials [Internet]. FratiniVergano – European Lawyers; 2025 [updated 13 January 2025. Available from: https://www.lexology.com/library/detail.aspx?g=34f19b17-1f1c-4500-9b11-a37251a468b7.
26. EFSA Panel on Food Contact Materials E, Aids P, Lambré C, Barat Baviera JM, Bolognesi C, Chesson A, et al. Re-evaluation of the risks to public health related to the presence of bisphenol A (BPA) in foodstuffs. EFSA Journal. 2023;21(4):e06857.
27. European Food Safety Authority, Agency EM. Report on divergent views between EFSA and EMA on EFSA's updated bisphenol A assessment. Joint report under Article 30 of Regulation (EC) No 178/2002. Parma and Amsterdam: European Food Safety Authority and European Medicines Agency; 2023 19 April 2023.
28. European Food Safety Authority, Assessment GFIfR. Report on diverging views between EFSA and BfR on EFSA updated bisphenol A assessment. Joint report under Article 30 of Regulation (EC) No 178/2002. Parma and Berlin: European Food Safety Authority and Bundesinstitut für Risikobewertung; 2023 19 April 2023.
29. Assessment GFIfR. Bisphenol A: BfR proposes health based guidance value, current exposure data are needed for a full risk assessment. BfR Opinion. Berlin: German Federal Institute for Risk Assessment (BfR); 2023 19 April 2023. Report No.: BfR Opinion No 018/2023.
30. Liu C, Duan W, Li R, Xu S, Zhang L, Chen C, et al. Exposure to bisphenol A disrupts meiotic progression during spermatogenesis in adult rats through estrogen-like activity. Cell death & disease. 2013;4(6):e676-e.
31. Srivastava S, Gupta P. Alteration in apoptotic rate of testicular cells and sperms following administration of Bisphenol A (BPA) in Wistar albino rats. Environmental Science and Pollution Research. 2018;25(22):21635-43.

32. Authority EFS, FitzGerald R, Loveren HV, Civitella C, Castoldi AF, Bernasconi G. Assessment of new information on Bisphenol S (BPS) submitted in response to the Decision 1 under REACH Regulation (EC) No 1907/2006. Wiley Online Library; 2020. Report No.: 2397-8325.
33. Wu N, He Y, Sun Z, Zhang S, Yang X, Liu QS, et al. The environmental occurrence, human exposure, and toxicity of novel bisphenol S derivatives: A review. Ecotoxicology and Environmental Safety. 2025;296:118182.
34. Commission E. Commission Regulation (EU) No 10/2011 of 14 January 2011 on plastic materials and articles intended to come into contact with food. Off J Eur Union. 2011;L 12:1-89.
35. Oh J, Choi JW, Ahn Y-A, Kim S. Pharmacokinetics of bisphenol S in humans after single oral administration. Environment international. 2018;112:127-33.
36. Safety SCoC. Opinion on Methylparaben (CAS No. 99-76-3, EC No. 202-785-7). SCCS final opinion, corrigendum of 28 February 2024. Luxembourg: European Commission, Directorate-General for Health and Food Safety; 2024 28 February 2024. Report No.: SCCS/1652/23.
37. Products SCoC. Extended Opinion on the Safety Evaluation of Parabens. SCCP opinion, adopted by written procedure 28 January 2005. Brussels: European Commission, Health & Consumer Protection Directorate-General, Directorate C – Public Health and Risk Assessment; 2005 28 January 2005. Report No.: SCCP/0873/05.
38. Authority EFS. Opinion of the Scientific Panel on food additives, flavourings, processing aids and materials in contact with food (AFC) related to para hydroxybenzoates (E 214–219). EFSA Journal. 2004;2(9):83.
39. Hoberman AM, Schreur DK, Leazer T, Daston GP, Carthew P, Re T, et al. Lack of effect of butylparaben and methylparaben on the reproductive system in male rats. Birth Defects Research Part B: Developmental and Reproductive Toxicology. 2008;83(2):123-33.
40. Matthews C, Davidson J, Bauer E, Morrison JL, Richardson AP. p-Hydroxybenzoic acid esters as preservatives II.: Acute and chronic toxicity in dogs, rats, and mice. Journal of the American Pharmaceutical Association (Scientific ed). 1956;45(4):260-7.
41. Oishi S. Lack of spermatotoxic effects of methyl and ethyl esters of p-hydroxybenzoic acid in rats. Food and chemical toxicology. 2004;42(11):1845-9.
42. Moos RK, Angerer J, Dierkes G, Brüning T, Koch HM. Metabolism and elimination of methyl, iso- and n-butyl paraben in human urine after single oral dosage. Archives of toxicology. 2016;90(11):2699-709.
43. Safety SCoC. Opinion on Propylparaben (PP). SCCS final opinion, adopted at plenary 30–31 March 2021. Luxembourg: European Commission, Directorate-General for Health and Food Safety; 2021 31 March 2021. Report No.: SCCS/1623/20.
44. Snodin D. Regulatory risk assessments: Is there a need to reduce uncertainty and enhance robustness? Update on propylparaben in relation to its EU regulatory status. Human & Experimental Toxicology. 2017;36(10):1007-14.
45. European Medicines Agency, Use CfMPfH. Reflection paper on the use of methyl- and propylparaben as excipients in human medicinal products for oral use. CHMP reflection paper, adopted 22 October 2015. London: European Medicines Agency; 2015 22 October 2015. Report No.: EMA/CHMP/SWP/272921/2012.
46. Fayyaz S, Kreiling R, Sauer UG. Application of grouping and read-across for the evaluation of parabens of different chain lengths with a particular focus on endocrine properties. Archives of Toxicology. 2021;95(3):853-81.
47. Sivaraman L, Pouliot L, Wang B, Brodie T, Graziano M, McNerney ME. Safety assessment of propylparaben in juvenile rats. Regulatory Toxicology and Pharmacology. 2018;92:370-81.
48. Shin M-Y, Shin C, Choi JW, Lee J, Lee S, Kim S. Pharmacokinetic profile of propyl paraben in humans after oral administration. Environment international. 2019;130:104917.
49. Zahra Z, Song M, Habib Z, Ikram S. Advances in per-and polyfluoroalkyl substances (PFAS) detection and removal techniques from drinking water, their limitations, and future outlooks. Emerging Contaminants. 2025;11(1):100434.
50. Agency USEP. PFAS National Primary Drinking Water Regulation: General Fact Sheet. Fact sheet. Washington, DC: US Environmental Protection Agency, Office of Water; 2024 April 2024.

51. United States Environmental Protection Agency, Water Oo. Technical Fact Sheet: Drinking Water Health Advisories for Four PFAS (PFOA, PFOS, GenX chemicals, and PFBS). Technical fact sheet. Washington, DC: US Environmental Protection Agency, Office of Water; 2022 June 2022. Report No.: EPA 822-F-22-002.
52. United States Environmental Protection Agency, Water Oo. Drinking Water Health Advisory: Perfluorobutane Sulfonic Acid (CASRN 375-73-5) and Related Compound Potassium Perfluorobutane Sulfonate (CASRN 29420-49-3). Final drinking water health advisory. Washington, DC: US Environmental Protection Agency, Office of Science and Technology, Health and Ecological Criteria Division; 2022 June 2022. Report No.: EPA/822/R-22/006.
53. Chain EPoCitF, Schrenk D, Bignami M, Bodin L, Chipman JK, del Mazo J, et al. Risk to human health related to the presence of perfluoroalkyl substances in food. Efsa Journal. 2020;18(9):e06223.
54. Lambert JC, Oesterling EO, Angrish M, Arzuaga X, Congleton J, Druwe I, et al. Provisional Peer-Reviewed Toxicity Values for Perfluorobutane Sulfonic Acid (CASRN 375-73-5) and Related Compound Potassium Perfluorobutane Sulfonate (CASRN 29420-49-3). Provisional Peer-Reviewed Toxicity Value (PPRTV) assessment. Cincinnati, OH: U.S. Environmental Protection Agency, Office of Research and Development, Center for Public Health and Environmental Assessment; 2021 April 2021. Report No.: EPA/690/R-21/001F.
55. Feng X, Cao X, Zhao S, Wang X, Hua X, Chen L, Chen L. Exposure of pregnant mice to perfluorobutanesulfonate causes hypothyroxinemia and developmental abnormalities in female offspring. Toxicological Sciences. 2017;155(2):409-19.
56. Abraham K, Mertens H, Richter L, Mielke H, Schwerdtle T, Monien BH. Kinetics of 15 per- and polyfluoroalkyl substances (PFAS) after single oral application as a mixture - A pilot investigation in a male volunteer. Environ Int. 2024;193:109047.
57. Abraham K, Gürtler R, Berg K, Heinemeyer G, Lampen A, Appel KE. Toxicology and risk assessment of 5-Hydroxymethylfurfural in food. Mol Nutr Food Res. 2011;55(5):667-78.
58. Farag MR, Alagawany M, Bin-Jumah M, Othman SI, Khafaga AF, Shaheen HM, et al. The Toxicological Aspects of the Heat-Borne Toxicant 5-Hydroxymethylfurfural in Animals: A Review. Molecules. 2020;25(8).
59. Laddha AP, Murugesan S, Kulkarni YA. In-vivo and in-silico toxicity studies of daidzein: an isoflavone from soy. Drug Chem Toxicol. 2022;45(3):1408-16.
60. Multigenerational reproductive study of genistein (Cas No. 446-72-0) in Sprague-Dawley rats (feed study). Natl Toxicol Program Tech Rep Ser. 2008(539):1-266.
61. Safety SCoC. Opinion on Genistein and Daidzein. SCCS final opinion, corrigendum of 11 October 2022. Luxembourg: European Commission, Directorate-General for Health and Food Safety; 2022 11 October 2022. Report No.: SCCS/1641/22.
62. Lewis RW, Brooks N, Milburn GM, Soames A, Stone S, Hall M, Ashby J. The effects of the phytoestrogen genistein on the postnatal development of the rat. Toxicol Sci. 2003;71(1):74-83.
63. Retana-Márquez S, Juárez-Rojas L, Hernández A, Romero C, López G, Miranda L, et al. Comparison of the effects of mesquite pod and Leucaena extracts with phytoestrogens on the reproductive physiology and sexual behavior in the male rat. Physiol Behav. 2016;164(Pt A):1-10.
64. Haque MU, Alam AHMK, Rahman MAA, Islam MS, Jibon MDK, Sujon KM, et al. Comprehensive pharmacological evaluation of protocatechuic acid methyl ester from Bombax ceiba as a novel anti-inflammatory and antitumor agent: Insights from in vivo efficacy and in silico studies. Results in Chemistry. 2025;18:102694.

Chapter S4 - Clinical characteristics of the study cohort and statistical comparisons

Clinical Characteristics According to Parenteral Nutrition

	No Parenteral Nutrition (n = 8)	Parenteral Nutrition (n = 13)
Sex (male/female)	4/4	7/6
Gestational age (weeks)	26.2 ± 0.9	25.7 ± 1.4
Birth weight (g)	774 ± 109	787 ± 178
APGAR score at min 5 (min-max)	4–9	7–9
APGAR score at min 10 (min-max)	4–9	9–9
Retinopathy of prematurity (%)	37.5%	61.5%
Bronchopulmonary dysplasia (%)	25%	46.2%
Necrotizing enterocolitis (%)	0%	15.4%
Total number of pRBC transfusions (mean / min - max)	2 / 0-4	8 / 2-16
Respiratory support at sampling (%)	MV: 0% | nCPAP: 37.5% | HFNC: 62.5%	MV: 15.4% | nCPAP: 61.5% | HFNC: 23.1%

Data are presented as mean ± SD unless otherwise indicated. APGAR scores are presented as minimum–maximum; sex as male/female counts; categorical variables as percentages.

Abbreviations: HFNC - high-flow nasal cannula; MV - mechanical ventilation; nCPAP - nasal continuous positive airway pressure; pRBC - packed red blood cells.

Wilcoxon rank-sum comparison of xenobiotic exposure according to parenteral nutrition

Analyte	No Parenteral Nutrition n = 8	Parenteral Nutrition n = 13	Wilcoxon Statistic	p-value	Adjusted p-value
HMFA	8	13	12	0.002	**0.010**
BBP	8	13	75	0.100	0.250
MEHP	8	13	46	0.690	0.690
BPA	8	13	44	0.587	0.690
NBBS	8	13	62.5	0.469	0.690

Wilcoxon rank-sum test. P-values were BH-corrected for multiple comparisons; adjusted p <0.05 was considered significant.

Sensitivity analysis of xenobiotic exposure according to parenteral nutrition

Infants who received red blood cell transfusions within 3 days prior to sampling were excluded from analysis

Analyte	No Parenteral Nutrition (n)	Parenteral Nutrition (n)	Wilcoxon Statistic	p-value	Adjusted p-value
HMFA	8	7	8	0.007	**0.036**
BBP	8	7	40.5	0.163	0.408
MEHP	8	7	27	0.954	0.954
BPA	8	7	23	0.602	0.752
NBBS	8	7	37.5	0.297	0.495

Sensitivity analysis excluding infants who received packed red blood cell (pRBC) transfusion within 3 days prior to sampling. Wilcoxon rank-sum tests were performed. P-values were adjusted using the Benjamini-Hochberg (BH) method.

Correlation of HMFA with xenobiotics in plasma of infants receiving parenteral nutrition

Analyte	n	Pearson Correlation Coefficient (*r*)	p-value	Adjusted p-value
BBP	13	0.08	0.798	0.798
BPA	13	0.67	0.012	**0.024**
MEHP	13	0.71	0.006	**0.024**
NBBS	13	0.61	0.026	**0.035**

Pearson correlations were calculated using log10-transformed concentrations. P-values were adjusted for multiple comparisons using the Benjamini-Hochberg method.

Correlation of HMFA with xenobiotics in plasma of infants irrespective of PN status

Analyte	n	Pearson Correlation Coefficient (r)	p-value	Adjusted p-value
BBP	21	-0.11	0.623	0.623
BPA	21	0.47	0.032	0.064
MEHP	21	0.60	0.004	**0.016**
NBBS	21	0.29	0.208	0.277

Pearson correlations were calculated using log10-transformed plasma concentrations. P-values were adjusted for multiple comparisons using the Benjamini-Hochberg method.
